# Sporangiospore Size Dimorphism Is Linked to Virulence of *Mucor circinelloides*

**DOI:** 10.1371/journal.ppat.1002086

**Published:** 2011-06-16

**Authors:** Charles H. Li, Maria Cervantes, Deborah J. Springer, Teun Boekhout, Rosa M. Ruiz-Vazquez, Santiago R. Torres-Martinez, Joseph Heitman, Soo Chan Lee

**Affiliations:** 1 Department of Molecular Genetics and Microbiology, Duke University Medical Center, Durham, North Carolina, United States of America; 2 Departamento de Genetica y Microbiologia, Facultad de Biologia, Universidad de Murcia, Murcia, Spain; 3 CBS-KNAW Fungal Biodiversity Centre, Utrecht, The Netherlands; University of Wisconsin-Madison, United States of America

## Abstract

*Mucor circinelloides* is a zygomycete fungus and an emerging opportunistic pathogen in immunocompromised patients, especially transplant recipients and in some cases otherwise healthy individuals. We have discovered a novel example of size dimorphism linked to virulence. *M. circinelloides* is a heterothallic fungus: (+) *sex* allele encodes SexP and (−) *sex* allele SexM, both of which are HMG domain protein sex determinants. *M. circinelloides* f. *lusitanicus* (*Mcl*) (−) mating type isolates produce larger asexual sporangiospores that are more virulent in the wax moth host compared to (+) isolates that produce smaller less virulent sporangiospores. The larger sporangiospores germinate inside and lyse macrophages, whereas the smaller sporangiospores do not. *sexMΔ* mutants are sterile and still produce larger virulent sporangiospores, suggesting that either the *sex* locus is not involved in virulence/spore size or the *sexP* allele plays an inhibitory role. Phylogenetic analysis supports that at least three extant subspecies populate the *M. circinelloides* complex in nature: *Mcl*, *M. circinelloides* f. *griseocyanus*, and *M. circinelloides* f. *circinelloides* (*Mcc*). *Mcc* was found to be more prevalent among clinical *Mucor* isolates, and more virulent than *Mcl* in a diabetic murine model in contrast to the wax moth host. The *M. circinelloides sex* locus encodes an HMG domain protein (SexP for plus and SexM for minus mating types) flanked by genes encoding triose phosphate transporter (TPT) and RNA helicase homologs. The borders of the *sex* locus between the three subspecies differ: the *Mcg sex* locus includes the promoters of both the TPT and the RNA helicase genes, whereas the *Mcl* and *Mcc sex* locus includes only the TPT gene promoter. Mating between subspecies was restricted compared to mating within subspecies. These findings demonstrate that spore size dimorphism is linked to virulence of *M. circinelloides* species and that plasticity of the *sex* locus and adaptations in pathogenicity have occurred during speciation of the *M. circinelloides* complex.

## Author Summary

Mucormycosis is recognized as an emerging infectious disease. Compared to other fungal infections, mucormycosis results in high mortality: ∼50% of overall infections and >90% in disseminated infections. There is therefore an ongoing need to study these fungal pathogens. However, surprisingly little is known about the pathogenesis of mucormycosis. Our findings reveal a correlation between sporangiospore size and virulence: larger sporangiospores are more virulent than small spores. Larger spores start invasive hyphal growth immediately upon phagocytosis by host immune cells, whereas smaller spores have a long period of isotropic growth. Differential host immune response explains their difference in virulence, whereby larger spores escape host immune cells by germinating inside of macrophages. Our findings revealed an example of adaptation through fungal cell gigantism, which enable pathogenic fungi to survive within and establish infection in the host. Knowledge of the mechanisms of pathogenicity and the molecular basis of the sexual cycle in *M. circinelloides* will contribute to advance our understanding of pathogenic zygomycetes.

## Introduction

Zygomycetes and chytridiomycetes are basal lineages of the fungal kingdom and both are paraphyletic and encompass several phyla [1], [2]. Within the Zygomycota, the order Mucorales is a monophyletic group that has been relatively well studied compared to other basal fungal groups. However, molecular data and our knowledge of sex and virulence in this fungal lineage is still limited.

*M. circinelloides* belongs to the order Mucorales and is a dimorphic fungus that grows as a budding yeast anaerobically and as a filamentous fungus aerobically [3], [4]. *M. circinelloides* is a causal agent for the rare but lethal fungal infection mucormycosis (also known as zygomycosis). Mucormycosis is an emerging infectious disease [5], [6] and is recognized as a prevalent fungal infection in patients with impaired immunity [7]. Recent data indicate a significant increase in mucormycosis due to an increasing population of immunocompromised patients with, for example, diabetes or AIDS, hematologic malignancies, hematopoietic stem cell/solid organ transplantation, or trauma [7]–[11]. High serum iron levels are also a risk factor that increases susceptibility to mucormycosis [7], [8], [12], and the high affinity iron permease, Ftr1, is known to be a virulence factor in the zygomycete *Rhizopus oryzae* in a murine host model [13]. Recently the host endothelial cell receptor GRP78 was shown to be overexpressed during *R. oryzae* infection in human umbilical vein endothelial cells, resulting in increased susceptibility to mucormycosis [14].

A major concern with mucormycosis is the high mortality rate, which is ∼50% in general and >90% in disseminated infections [8]–[10], [12], [15]. Causal agents are zygomycetes including *Rhizopus* spp., *Mucor* spp., *Rhizomucor*, *Absidia* spp., *Cunninghamella* spp., among others [6], [16]. A recent study found that in 50 cases in solid organ transplant recipients *Mucor* spp. caused 37% of mucormycosis cases followed by *Rhizopus* spp (35%) and *Mycocladus* (13%) [17]. In a European survey of 230 mucormycosis cases, *Rhizopus* spp. caused 24% of the cases, followed by *Mucor* spp. (22%) and *Absidia* spp. (14%) [18]. The predicted economic burden in the US health care system caused by mucormycosis is ∼$100,000 per case [19]. However, surprisingly little is known about the genetics of pathogenesis for zygomycetes compared to other fungal pathogens [20].

*M. circinelloides* is a heterothallic [(+) and (−) strains] zygomycete and propagates through both asexual and sexual life cycles. In the asexual life cycle, spores germinate and undergo hyphal growth, and complex mycelia are formed, from which aerial hyphae form culminating at their apices in sporangia harboring multinucleate asexual spores (sporangiospores). In the sexual life cycle, hyphae from the two different mating types recognize each other and then fuse to form zygospores in which meiosis occurs. The zygospores later send a hypha to produce a sporangium containing meiospores at the apex. Sexual development is mediated by a zygomycete specific pheromone, trisporic acid. Minus (−) and plus (+) mating types secrete and exchange trisporic acid precursors that are converted in the opposite mating type to mature trisporic acid [21]. Trisporic acid triggers the formation of specialized hyphae (zygophores) supporting the zygospores followed by hyphal fusion of the opposite mating types to form a zygote. Meiosis then occurs.

The *sex* locus of zygomycetes, including *M. circinelloides*, *Phycomyces blakesleeanus*, and *R. oryzae*, governs and orchestrates sexual reproduction and consists of a high mobility group (HMG) transcription factor gene flanked by genes encoding a triose phosphate transporter homolog (TPT) and an RNA helicase [22]–[24]. The HMG domain proteins are designated SexP for the (+) and SexM for the (−) mating types. The sequences of the genes encoding SexP and SexM are divergent but allelic in the (+) and (−) mating types, in contrast to the idiomorphic nature of *MAT* in many ascomycetes and basidiomycetes encoding entirely divergent proteins [25].

The evolutionary trajectory of sex in fungi is an intriguing subject, and provides a forum to elucidate the basis of sexual development and the evolution of sex [26]. For example, complete genome sequences of several pathogenic and non-pathogenic *Candida* species revealed a dramatic divergence of *MAT* loci and sexuality in the *Candida* clade [26]–[28]. The studies reveal that sexual development and its specification are differentially adapted in each species. Additionally, *Cryptococcus* species were also found to be divergent in *MAT* locus structure and sexuality [26], [29], [30]. In contrast to the bipolar species *C. neoformans* and *C. gattii*, *Cryptococcus heveanensis* retains a sexual cycle involving a tetrapolar system with unfused gene clusters, one containing the homeodomain genes and the other pheromone/pheromone receptor genes [29]. Within the *Cryptococcus* lineage, *C. heveanensis* represents an evolutionary intermediate in the trajectory from a tetrapolar to a bipolar mating system.

The *M. circinelloides* complex has been characterized based on physiological characteristics and includes *M. circinelloides* f. *lusitanicus* (*Mcl*), *M. circinelloides* f. *griseocyanus* (*Mcg*), and *M. circinelloides* f. *circinelloides* (*Mcc*) [31]. In this study, we examined the mating, virulence, and *sex* locus of the three subspecies of *M. circinelloides*. Multi-locus sequence typing (MLST) was applied to construct a phylogeny of the *M. circinelloides* subspecies complex. We also tested the virulence of each subspecies in larvae of the wax moth, *Galleria mellonella*, a heterologous host model; a significant difference in virulence in the *M. circinelloides* subspecies was observed. Spore size was found to be correlated with virulence in that larger spores of (−) mating type were found to be more virulent than smaller spores of (+) mating type. We disrupted the *sexM* gene in the (−) mating type, and found that *sexMΔ* mutants are sterile in genetic crosses, functionally verifying a key role in sex determination and sexual development for the first time in this basal fungal phylum. The virulence analysis was extended to a diabetic murine host model including analysis of clinical *M. circinelloides* isolates, revealing the *Mcc* subspecies is highly virulent in mice which is well correlated with its more frequent occurrence in human clinical isolates. A comparison of the *sex* loci between the subspecies is presented here and the evolutionary trajectory of the *sex* locus in the *M. circinelloides* complex is posited. Expansion of the *sex* locus in one subspecies of *M. circinelloides* into the RNA helicase promoter region contrasts with the *sex* locus of two related zygomycetes, *P. blakesleeanus* and *R. oryzae*, and reveals the evolutionary plasticity of this dynamic region of the genome involving either expansion or contraction.

## Results

### Sporangiospore size as a virulence factor

**Figure 1:**
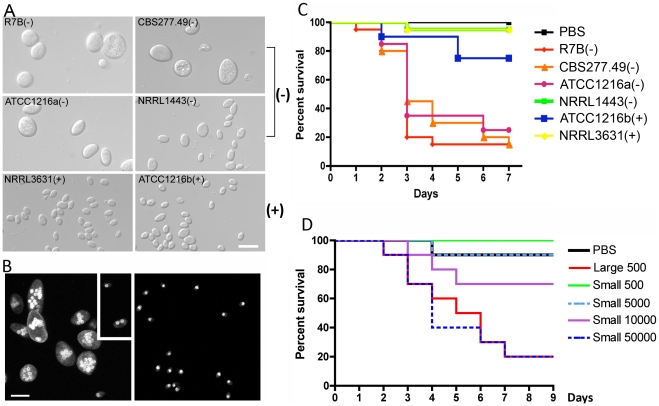
Correlation between spore size and virulence in *M. c.* f. *lusitanicus*. (A) Several strains of *Mcl* display differences in spore size. Two (−) strains (R7B and ATCC1216a) produce larger spores (12.32±2.71 µm long for R7B), and two (+) strains (NRRL3631 and ATCC1216b) produce smaller spores (4.70±0.91 µm long for NRRL3631). One (−) isolate, NRRL1443, produces intermediate sized spores. Note that R7B is an auxotrophic mutant (leucine^−^) of CBS277.49 and the mutation in the *leuA* gene did not impact virulence. (B) Nuclei were stained with DAPI and cell walls were stained with calcofluor. Combined Z-stack images show a difference in the number of nuclei in the (−) and (+) mating type spores. The left panel shows multinucleated spores; however, smaller spores of the (−) mating type (small rectangular area) are uni- or bi-nucleate. The (+) spores in the right panel are uninucleate. Scale = 10 µm. N.A. = 1.4 with oil. (C) Virulence in the wax moth host is correlated with spore size. Three (−) strains (R7B, CBS277.49, and ATCC1216a) are significantly more virulent compared to the (+) strains with a smaller spore size (see the text for statistics). (D) In the survival curves of wax moth larvae infected with different numbers of (+) spores (NRRL3631), only 50,000 (+) spores are similarly virulent to 500 (−) spores (R7B) (*p* = 0.8826). See Figure 1S for the analysis of additional large (−) and small (+) sporangiospore producing strains further substantiating the link of spore size to virulence.

*M. circinelloides* is recognized as an agent responsible for mucormycosis, a rare fungal infection associated with a high mortality rate. The *Mucor circinelloides* complex is known to consist of three extant subspecies: *M. circinelloides* f. *lusitanicus* (*Mcl*), *M. circinelloides* f. *circinelloides* (*Mcc*), and *M. circinelloides* f. *griseocyanus* (*Mcg*) [31]. Among them, the genome of one of *Mcl* isolate, CBS277.49 (Table 1), has been sequenced (US Department of Energy Joint Genome Institute *M. circinelloides* genome project). Asexual sporangiospores are involved in dissemination, whereas sexual zygospores are considered to be dormant. Therefore, sporangiospores were tested in this study, in which spores indicate sporangiospores unless otherwise stated. We observed that in *Mcl* spore size and shape differ between (−) and (+) mating type isolates of *M. circinelloides* (Figure 1A). Minus (−) strains produce larger, irregularly shaped spores that are on average 12.3±2.7 µm, while (+) isolates are smaller in spore size (4.7±0.9 µm) and more homogenous in shape (Table 2). We further analyzed all currently available *Mcl* isolates (14 total) and found that asexual spores of (+) mating type isolates are homogenously smaller compared to those of (−) mating type isolates, in which three (−) isolates produce larger spores and the other 7 (−) mating type isolates produce spores of an intermediate size (Figure 1 and Figure S1). When visualized inside of the sporangia, the spores already exhibit a difference in size and shape (Figure S2).

**Table 1:** *Mucor circinelloides* strains used in this study.

Subspecies	Strains	Mating type
*Mucor circinelloides f. lusitanicus*	ATCC 90680 (R7B)	(−)
	NRRL 3631	(+)
	CBS 277.49	(−)
	ATCC 1216b	(+)
	NRRL 1443	(−)
	ATCC1216a	(−)
	CBS852.71*	(+)
	CBS847.72*	(−)
	CBS108.18*	(−)
	CBS108.19*	(−)
	CBS242.33*	(−)
	CBS253.35*	(−)
	CBS633.65*	(−)
	CBS968.68*	(+)
	CBS969.68*	(−)
*Mucor circinelloides f. circinelloides*	ATCC 1209b	(−)
	ATCC 11010	(−)
	NRRL 3615	(+)
	NRRL 3614	(−)
*Mucor circinelloides* f. *griseocyanus*	ATCC 1207b	(−)
	ATCC 1207a	(+)

Clinical *Mucor* isolates		
Subspecies	Strains	Source
*Mucor circinelloides f. circinelloides (Mcc)*	CBS195.68**	Air, Netherlands [69]
	IP1873.89	Human feces, France [69]
	CNRMA03.154	Human skin, France [69]
	CNRMA03.371	Human muscle, France [69]
	CNRMA04.805	Human muscle, France [69]
	Megoma	Transplant recipient [17]
	UIC-1	Transplant recipient [17]
	D-tate	Transplant recipient [17]
	AS71	Transplant recipient [17]

*Phylogenetic analysis with MLST confirmed that they are M. circinelloides f. lusitanicus. Mating type was confirmed by sexP/M specific primers and mating assays.

**Environmental isolate.

**Table 2:** *M. circinelloides* f. *lusitanicus* (−) isolates produce larger spores than (+) isolates and other subspecies.

Subspecies	Strain	Length (µm)	Width (µm)
*M. c.* f. *lusitanicus*	R7B (−)	12.32±2.71	8.85±2.2
	NRRL3631 (+)	4.7±0.91	3.52±0.72
*M. c.* f. *circinelloides*	NRRL3614 (−)	5.86±0.46	4.49±0.19
	NRRL3615 (+)	4.59±0.64	3.83±0.51
*M. c.* f. *griseocyanus*	ATCC1207b (−)	4.87±0.64	3.89±0.66
	ATCC1207a (+)	4.69±0.57	3.89±0.52

Average spore sizes (n = 100).

The nuclei of (−) and (+) spores were stained with DAPI and observed by confocal microscopy. Larger spores contain more nuclei than smaller spores, where larger (−) spores contain multiple nuclei (from 1 to 16) whereas smaller (+) spores are uniformly uninucleate (Figure 1B). The (−) spore population is also mixed with a minority of smaller spores with sizes comparable to those of the (+) isolates. Interestingly, these smaller (−) spores contain fewer nuclei (1 or 2) compared to larger (−) spores (Figure 1B).

When we tested the virulence of *Mcl* strains in a heterologous host, *Galleria mellonella*, which has been used as a host for several human fungal pathogens and pathogenic bacteria ([32], [33] and references therein), a correlation between larger spore size and enhanced virulence was apparent (Figure 1C and Figure S1). Five hundred sporangiospores of each strain were suspended in PBS and injected through the pseudopod of the wax moth larvae. We monitored the viability of infected larvae at one day intervals. Interestingly, strains with larger spores were more virulent than ones with smaller spores; for example R7B(−) was significantly more virulent than NRRL3631(+) (*p*<0.0001); however, NRRL3631(+) was not significantly virulent compared to the PBS control (*p* = 0.3173). Intermediate sized spores of NRRL1443(−) showed no significant virulence in comparison with PBS or (+) strains [*p* = 0.3173 for NRRL1443(−) vs PBS, *p* = 1.0000 for NRRL1443(−) vs NRRL3631(+)]. When nine additional *Mcl* isolates were tested for virulence in the wax moth host, the larger spore producing isolates were all more virulent, further substantiating the conclusion that larger spore isolates are more virulent than smaller spore producing isolates (Figure S1). These results provide evidence that spore size dimorphism is an important virulence factor in the invertebrate model.

Based on these findings, we propose two possible hypotheses: 1) fungal biomass could affect virulence, wherein the (−) spores challenge the host with more fungal material compared to the (+) spores, or 2) the host may respond differently to larger spores. To test these hypotheses, we examined pathogenesis in the wax moth model with increased numbers of (+) spores (Figure 1D). In this experiment, 50,000 (+) spores displayed similar virulence to 500 larger (−) spores (*p* = 0.8826). Furthermore significantly more (+) spores (10,000) were less virulent than 500 (−) spores (*p* = 0.0421). With the assumption that the spores are spherical, the larger spores are ∼18 times greater in volume than the smaller spores. Our virulence test indicates that a 20-fold numerical excess of smaller spores is less virulent than the larger spores (despite the roughly equivalent biomass of the two inocula), and a 100-fold numerical excess of smaller spores is required for equivalent virulence to the larger spores, suggesting that the first hypothesis about the possible effect of biomass is not sufficient to explain the marked difference in virulence of (−) vs. (+) spores.

**Figure 2:**
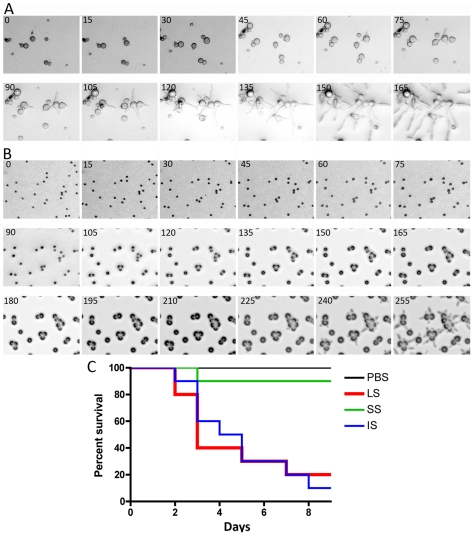
Delay in isotropic to polarized growth transition during germination of small vs. large spores. (A) Large spores (R7B) have a very short isotropic growth stage or bypass it entirely to send germ tubes. (B) Small spores (NRRL3631) grow isotropically to reach the size comparable to that of large spores, and then germ tubes emerge. Time-lapse (every ∼15 min) images of each strain are presented. Note that in panel B, there are 3 rows of images to show the delay (∼225 mins) prior to germ tube emergence in the smaller (+) spores. See Videos S1 and S2 for corresponding videos. Scale = 40 µm. (C) When isotropically grown, the enlarged (+) spores became as virulent as larger spores in the wax moth larva host (*p* = 0.9878).

We observed and analyzed the germination of large and small spores (Figure 2A and B, Videos S1 and S2). Interestingly, the larger spores display a shorter isotropic growth phase or bypass the isotropic growth stage resulting in a rapid and immediate germ tube emergence after exiting dormancy. In constrast, the smaller (+) spores grow isotropically for a longer time until their size is comparable to that of the larger (−) spores, and they then start sending germ tubes. This difference in germination kinetics between the larger and smaller spores may contribute to the differences in their virulence. To address this, smaller spores were grown isotropically and then used to infect wax moth larvae to test the effect on virulence. Smaller spores were grown in liquid YPD media for 3.5 hours until they attained a size comparable to the larger spores. We found that the isotropic growth of small spores yields large multinucleate spores similar to (−) larger spores (data not shown). These spores were collected for inoculation and 1,000 each of the larger (−) spores (LS), smaller (+) spores (SS), and isotropically grown (+) spores (IS) were injected into ten wax moth larvae and survival was monitored. Interestingly IS are as virulent as LS (Figure 2C). These findings further support the conclusion that spore size is a virulence factor.

**Figure 3:**
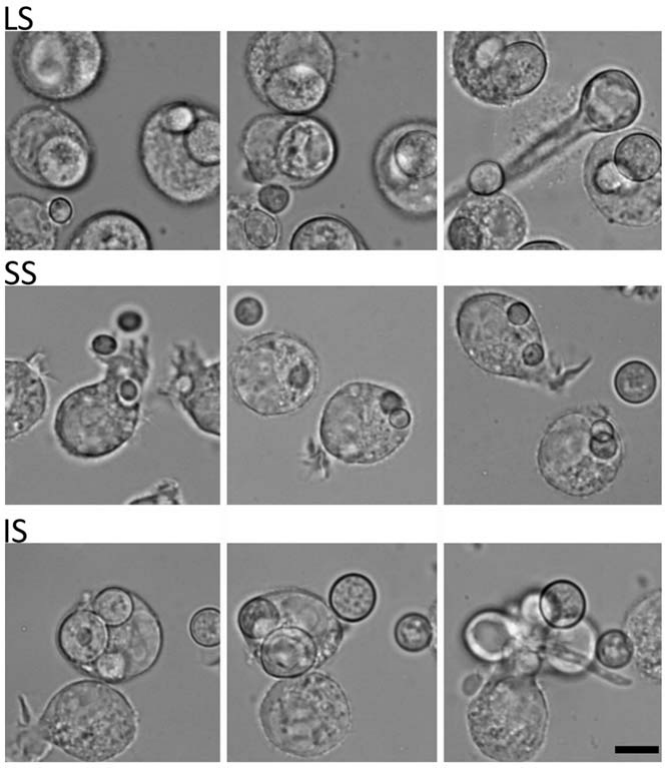
Time-lapse analysis of response of the murine macrophage to large spores (LS), small spores (SS), and isotropically grown spores (IS). The macrophage (J774) engulfs each of the different-sized spores successfully. Both LS and IS germinate inside macrophages indicating that their invasive hyphal growth is not inhibited by the macrophage. In contrast, SS are trapped inside the macrophage and either do not undergo isotropic growth or exhibit very limited growth when compared to the spores outside of the macrophage that grow isotropically. Time progresses across each row in the images from left to right.

The host response to different sized spores is of interest to consider. We observed that cultured murine macrophage cells (J774) respond differently to LS, SS, and IS (Figure 3 and Videos S3, S4, and S5). When co-cultured, spores of all sizes were phagocytosed by macrophages. A characteristic difference is that the larger spores germinated inside of the macrophages, whereas smaller spores remained dormant inside macrophages without isotropic growth or germination, and grew significantly more slowly compared to the small spores outside of macrophages. Interestingly, IS also germinated inside macrophages similar to the LS. Thus, LS and IS are both likely to undergo invasive hyphal growth in hosts and may therefore exhibit higher virulence. We also observed that, with longer co-incubation, macrophages harboring LS or SS or IS all underwent lysis (data not shown), which warrants further study to elucidate how macrophage cell lysis is triggered by the encounter with fungal spores.

### SEM and TEM analyses of the larger and smaller spores

**Figure 4:**
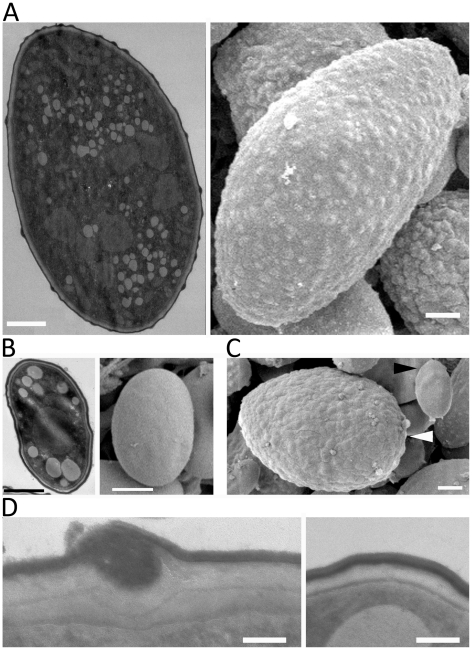
SEM and TEM images of large and small spores. (A) Large spores are decorated with ‘bumps’ on their surface. (B) The surface of small spores is smooth. (C) The subpopulation of small spores in the larger spore producing isolates (CBS277.49) has a smooth surface (black arrowhead) compared to larger spores (white arrowhead). (D) Trafficking to the cell wall is observed in the large spores (left) but not in the small spore (right) indicating that the larger spores may be metabolically active for further development, such as, invasive hyphal growth. Scales are 1 µm for A, B, and C, and 100 nm for D.

The difference in virulence and early germination prompted us to examine the detailed microscopic structure to assess differences between the larger and smaller spores. Interestingly, SEM analyses revealed that the larger (−) spores are decorated with ‘bumps’ on the surface, whereas the surface of the smaller (+) spores is smooth (Figure 4A and B). As described above, the (−) isolates producing larger spores also produce a subpopulation of smaller spores (Figure 1B). We also observed that small, uninucleate (−) spores have a smooth surface unlike the bumpy larger (−) spores (Figure 4C). Based on TEM, the spore surface bumps may result from trafficking processes from cytosol to the cell surface involved in cell wall construction (Figure 4D).

### *sexM* controls sexual reproduction but not spore size

**Figure 5:**
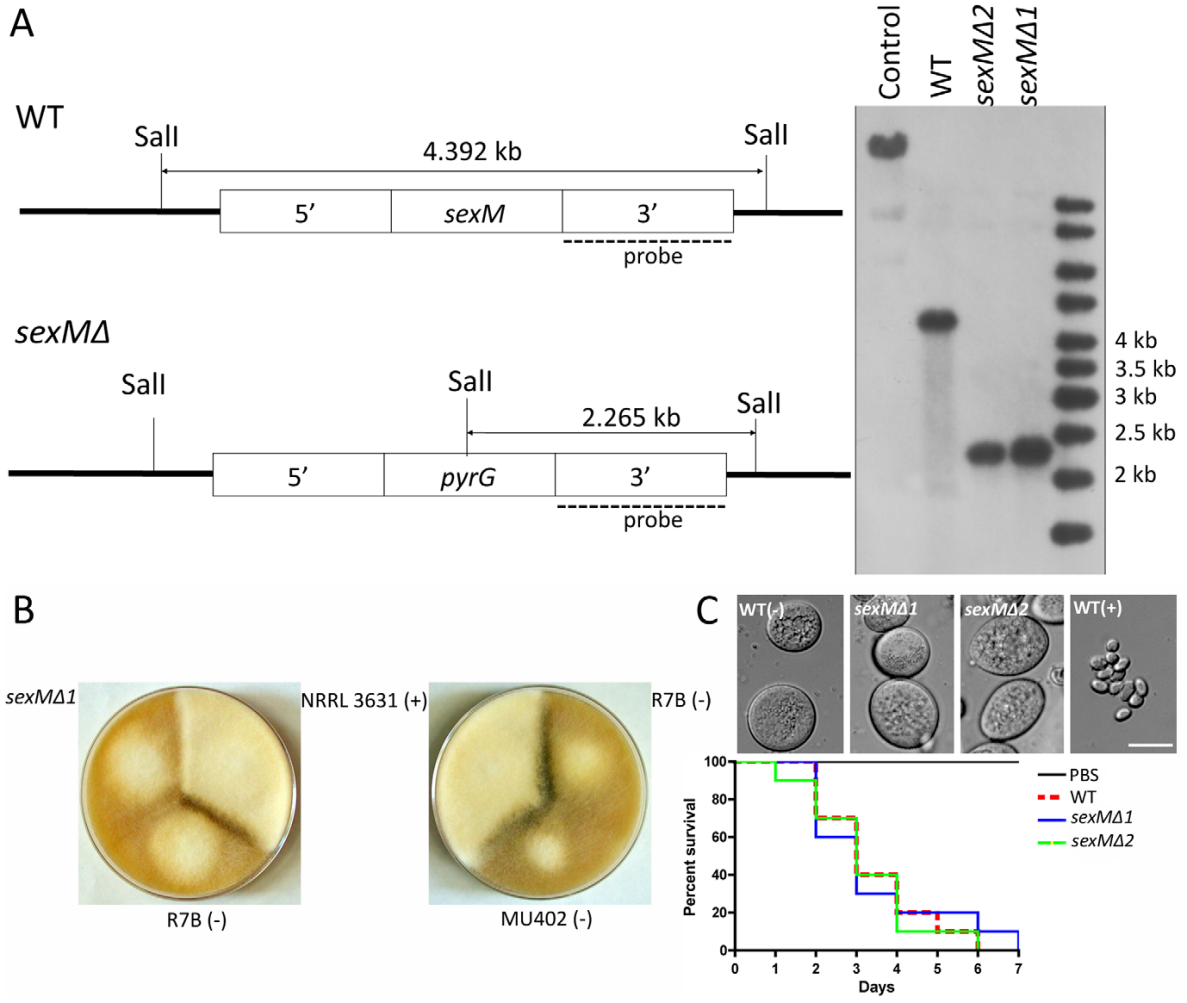
Disruption of the *sexM* gene and mating and virulence tests of the *sexM* mutants. (A) Southern blot analysis shows that the *sexM* gene was replaced with the *pyrG* gene resulting in disruption of the *sexM* gene in two transformants. The *sexM* probe corresponds to a 1.3-kb EcoRI fragment of the 3′ region of *sexM*. (B) Two *sexM* mutants [MU423 (*sexMΔ1*) and MU424 (*sexMΔ2*)] are sterile in mating assays with (+) or (−) strains (see also Figure 3S). The parental MU402 strain is a *pyrG leuA* double mutant. (C) The *sexM* mutants do not display differences in spore size or virulence in the wax moth host. See Figure 3S for additional PCR validation of the *sexM* mutant strains.

Two *sexM* mutants [MU423 (*sexMΔ1*) and MU424 (*sexMΔ2*)] were obtained by transformation and homologous recombination with the *pyrG* cassette flanked with sequences 5′ and 3′ end of the *sexM* gene ORF. To obtain the transformants, 50 µg of the *pyrG* cassette DNA was co-incubated with protoplasts of the MU402 strain (See Materials and Methods) [34]. A total of 25 *pyrG* ^+^ transformants were obtained, which were grown on selective medium for several vegetative cycles to obtain homokaryotic transformants. PCR analysis designed to distinguish homologous from ectopic integration indicated that two of these transformants amplified the expected fragments from the 5′ and 3′ flanking regions (Figure S3). The homologous replacement was confirmed through Southern blot hybridization with a probe homologous to the *sexM* gene that can discriminate between wild-type and mutant alleles (Figure 5A). The absence of the 4.39 kb SalI wild-type fragment in the *sexMΔ* mutants confirmed that the wild-type allele had been successfully replaced in all of the nuclei of these mutant strains.

We tested mating ability by co-inoculating spores of either mating type with the *sexMΔ* mutants on YPD or YXT media with incubation for two weeks in the dark at room temperature. The *sexMΔ* mutants failed to form zygospores in any combination of crosses with wild-type (+), wild-type (−), or the other *sexMΔ* mutant (Figure 5B and Figure S3). Moreover, no self-fertile development was observed, excluding models in which SexM represses sexual development. That two *sexMΔ* mutants are both sterile provides evidence that *sexM* is essential for mating. The *sexMΔ* mutants have no apparent difference in spore size compared to (−) wild-type isolates, and in virulence tests in wax moth larvae the *sexMΔ* mutants were as virulent as the (−) wild-type (*p* = 0.8235 and 0.8619 for MU423 and MU424, respectively) (Figure 5C). Thus, SexM does not appear to be involved in spore size determination or virulence in the wax moth model. Although the *sexM* gene is not involved in virulence, the successful disruption of the *sexM* gene and functional verification of a role for the *sex* locus in sexual reproduction in this basal fungal lineage is a major advance in our understanding of sex in the Zygomycota basal fungal lineage.

### Phylogeny of *Mucor circinelloides* subspecies

**Figure 6:**
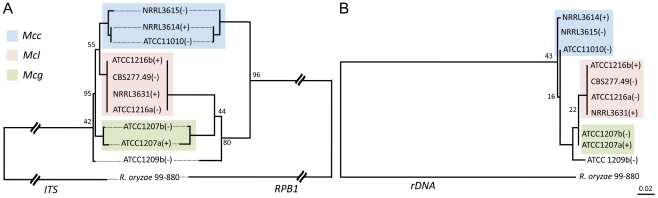
Phylogenetic relationships of the *M. circinelloides* subspecies. (A) *Mucor circinelloides* spp. compared with *R. oryzae* are shown using concatenated *RBP1*, *rDNA2*, and *ITS* loci. (B) Phylogenetic relationships constructed with the *RBP1*, *rDNA2*, and *ITS* loci in *Mcg*, *Mcc*, and *Mcl*. Both phylogenetic analyses provide evidence that there are three subspecies within the *M. circinelloides* complex. *Mcc*: *M. circinelloides* f. *circinelloides*, *Mcl*: *M. circinelloides* f. *lusitanicus*, and *Mcg*: *M. circinelloides* f. *griseocyanus*.

Previous characterization of the *Mucor circinelloides* subspecies complex was based on morphological and physiological characteristics [31]. To obtain a high-resolution phylogeny for the *M. circinelloides* subspecies (Table 1), a phylogenetic analysis based on multi-locus sequence typing (MLST) was performed with three of the genes analyzed in the fungal tree of life project [1]. These include an RNA polymerase subunit gene (*RBP1*), a large ribosomal RNA subunit gene (*rDNA2*), and one intragenic spacer region (*ITS*). All DNA sequences obtained were aligned and maximum likelihood trees were constructed for each of the three genes (Figure 6). Trees constructed with *RPB1*, *ITS*, and *rDNA2* sequences all revealed similar patterns, where three notable clusters are formed that correspond to the *M. circinelloides* f. *lusitanicus* (*Mcl*) (ATCC1216a, ATCC1216b, CBS277.49, NRRL3631), *M. circinelloides* f. *griseocyanus* (*Mcg*) (ATCC1207a, ATCC1207b), and *M. circinelloides* f. *circinelloides* (*Mcc*) (NRRL3614, NRRL3615, ATCC11010) subspecies. There was no phylogenetic incongruence observed demonstrating that the *M. circinelloides* subspecies are sufficiently diverged to be designated as at least subspecies. ATCC1209b was found to be distinct from all three subspecies based on this MLST analysis and may prove to be an intermediary, hybrid, or distinct subspecies within the *M. circinelloides* complex.

The clinical *Mucor circinelloides* isolates (Table 1) were classified at the subspecies level based on molecular analysis. Based on the previously analyzed MLST alleles (Figure 6), we found that most clinical strains (5 out of 8) grouped within the *Mcc* subspecies with the exception of AS71 and UIC-1 (Figure S4). These findings were consistent at all three MLST loci. In the phylogenetic analyses, we additionally found evidence for a fourth group containing ATCC1209b and UIC-1 (Table 1). These isolates clustered together in all three trees. ATCC1209b was found to be sterile with all other tested isolates (Table 2) and these two lines of evidence may indicate that ATCC1209b and UIC-1 could be isolates of a different subspecies.

### Virulence of the *Mucor circinelloides* subspecies complex

**Figure 7:**
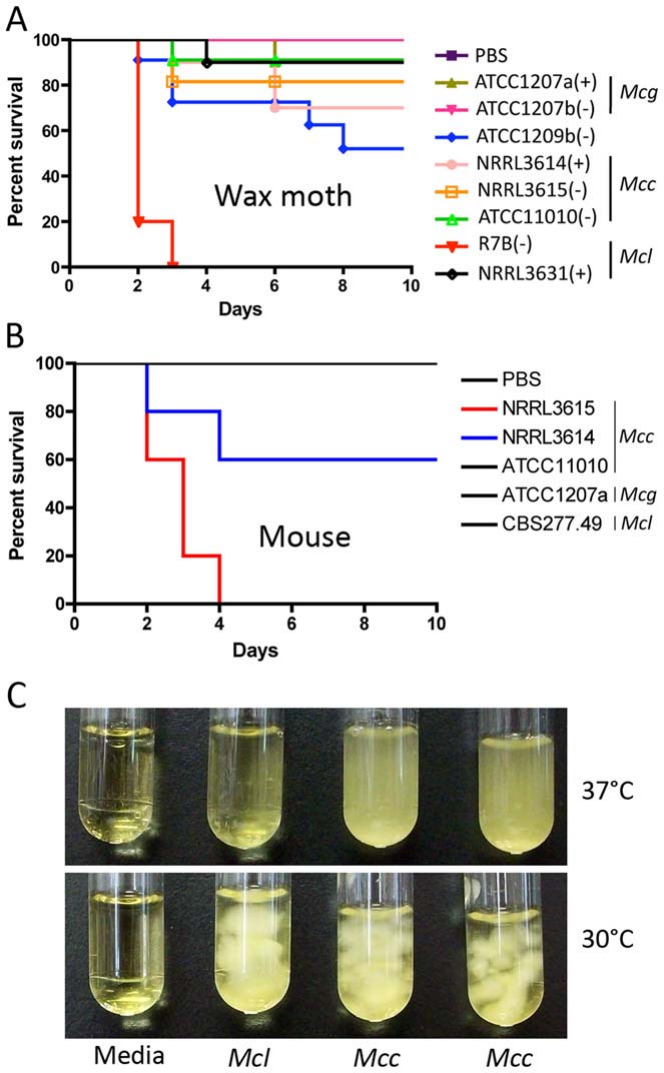
Virulence tests of the three *M. circinelloides* subspecies in the wax moth and murine hosts. (A) Larvae of the wax moth *Galleria mellonella* were used as the host. *Mcl* was found to be the most virulent, causing 100% mortality in 3 days. All other strains were less virulent. Injections were repeated three times with similar results. PBS injection served as a negative control. (B) Virulence of *M. circinelloides* subspecies in the murine host. Mice infected with NRRL3615 (*Mcc*) show 100% mortality in four days, and those with NRRL3614 (*Mcc*) show 40% mortality in four days. Both NRRL3615 and NRRL3614 are *Mcc* isolates that are commonly found in clinical isolates tested in this study. (C) *Mcc* (NRRL3614 and NRRL3615) exhibits more robust grows at 37°C compared to *Mcl* (CBS277.49), which may result in *Mcc* being highly virulent in the diabetic murine host compared to *Mcl*, unlike our observations in the wax moth host.

To elucidate possible differences in the pathogenicity between the *M. circinelloides* subspecies, we tested virulence using wax moth larvae. *M. c.* f. *lusitanicus* strain R7B was the most pathogenic (Figure 7A); for example, R7B caused 100% lethality within 3 days compared to 20% lethality with *M. c.* f. *griseocyanus* ATCC1207B over the course of 10 days (*p* value<0.001). All other strains tested were less virulent and a correlation between spore size and pathogenicity was observed (Table 2 and Figure 7).

Interestingly, although we observe greater virulence of *Mcl* in the wax moth, the *Mcc* subspecies is more common in human clinical cases. To assess whether the virulence of a given *M. circinelloides* subspecies in a mammalian host system differs from that observed in the wax moth heterologous host, we employed a diabetic mouse model ([14] and references therein) for zygomycosis. One million (1×10^6^) spores of *M. circinelloides* subspecies (Table 1) were intravenously inoculated into 5 mice for each strain. In this experiment, one *Mcc* species, NRRL3615, displayed the highest virulence (*p* = 0.0091) (Figure 7B) compared to *Mcl* and *Mcg*. The *Mcc* isolate NRRL3615 showed 100% mortality and NRRL3614 displayed 40% mortality by 4 days post infection. However the other *Mcc* isolate, ATCC11010, and *Mcl* species were avirulent. Thus, only *Mcc* isolates (but not all) show virulence in the murine host, which may explain the prevalence of *Mcc* species in clinical isolates. MU423 (*sexMΔ* mutant of *Mcl*), ATCC1207b (*Mcg*), and ATCC1209b were also tested and did not display mortality for the duration of the experiment (data not shown). More clinical isolates (Table 1) were tested in the murine host confirming that only *Mcc* isolates based on our phylogenetic analysis display virulence but less virulent *Mcc* strains were also found (Figures S4 and S5). Notably, we observed that the *Mcc* isolates exhibited better growth at 37°C compared to the other subspecies (Figure 7C) indicating that temperature sensitivity/resistance might contribute to the differences between *Mc* subspecies in virulence in the murine host.

### Mating of the *Mucor circinelloides* subspecies

**Figure 8:**
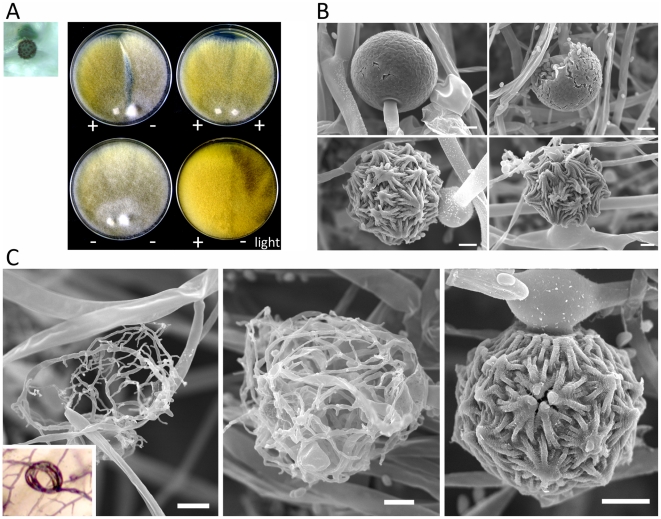
Sexual development of *Mucor circinelloides*. Light and scanning electron microscopy images were obtained with mounted samples of zygospores and other mating structures. (A) Images show a zygospore (enlarged insert) as viewed by light microscopy and the dark zygospore line that forms during mating. (+) and (−) designations indicate mating types of strains (ATCC1216a (−) and ATCC1216b (+) *Mcl* strains). A distinct dark zygospore line was found in (+)/(−) co-cultures but not in same-sex mating pairs. All matings were performed for 7 days in the dark with the exception of the bottom right plate that was incubated in the light at room temperature. Mating occurred in the dark and not in the light. (B) The sporangium (upper panels), the asexual spore harboring structure, and zygospores (lower panels), the sexual spores, are shown at higher magnification by SEM. (C) The formation of the zygospore structure is depicted by SEM. Zygospore formation is initiated by the production of coiled hyphae that entangle to form the mature zygospore. Coiled hyphae in a mating between ATCC1207a and ATCC1207b are presented in the small rectangular area (light microscopic image). Scale = 10 µm.

The mating of three *M. circinelloides* subspecies was examined in response to different conditions and media. Isolates from each *M. circinelloides* subspecies were co-cultured with each other (Table 3). Spores of each strain were inoculated onto PDA medium. After a 24-hour incubation, 5 mm×5 mm agar blocks containing mycelia of each strain were placed in abutting pairs on YXT media. The plates were wrapped with tin foil and incubated at room temperature or, in some cases, the mating plate was placed at a lower incubation temperature (see Materials and Methods). When co-cultured without light, a dark zygospore line formed in the middle between two opposing strains of opposite mating type (for example, ATCC1216a and ATCC1216b) (Figure 8A); however, two strains of the same mating type did not form zygospores based on morphological analysis by light microscopy. Zygospore formation was largely restricted to matings within a subspecies, with two notable exceptions in which zygospores were sporadically observed, involving the mating crosses of ATCC1207b (*Mcg*)×NRRL3615 (*Mcc*) and R7B (*Mcl*) and ATCC1207a (*Mcg*) (Table 3). Interestingly, the ATCC11010 *Mcc* isolate did not mate with any of the *Mcc* strains tested. The unclassified ATCC1209b isolate did not mate with any strain tested. Mating assays performed with the clinical isolates found evidence of mating in two isolates. Both CNRMA03.371 and CNRMA04.805 were found to mate with NRRL3614, indicating they are of the (+) mating type (data not shown). Crosses performed with other isolates did not reveal conclusive zygospore formation.

**Table 3:** Sexual reproduction of the *M. circinelloides* isolates.

		*Mcl*				*Mcc*			*Mcg*		
		R7B (−)	ATCC 1216a (−)	ATCC 1216b (+)	NRRL 3631 (+)	ATCC 11010 (−)	NRRL 3614 (−)	NRRL 3615 (+)	ATCC 1207b (−)	ATCC 1207a (+)	ATCC 1209b (−)
*Mcl*	R7B (−)	O	O	**Z**	**Z**	O	O	**C**	O	**Z**	O
	ATCC 1216a (−)		O	**Z**	**Z**	O	O	**C**	O	**C**	O
	ATCC 1216b (+)			O	0	**C**	**C**	O	**C**	O	**C**
	NRRL 3631 (+)				0	**C**	**C**	O	**C**	O	**C**
*Mcc*	ATCC 11010 (−)					O	O	**C**	O	**C**	O
	NRRL 3614 (−)						O	**Z**	O	**C**	O
	NRRL 3615 (+)							O	**Z**	O	**C**
*Mcg*	ATCC 1207b (−)								O	**Z**	O
	ATCC 1207a (+)									O	**C**
	ATCC 1209b (−)										O

(+) and (−) denote mating type of isolates.

Z indicates that zygospores were produced during co-culture, O indicates no zygospores produced.

‘C’ indicates potentially compatible co-cultures of (+) and (−) mating type strains without forming zygospores.

Zygospores of zygomycetes remain dormant for a long period from months to a year before germination occurs [21], [35]. *P. blakesleeanus* zygospores germinate after a 3 to 4 month dormancy period, enabling the analysis of progeny [23], [36], [37]. Although zygospore germination of other *Mucor spp.* under laboratory conditions has been reported [38]–[40], our extensive trials for *M. circinelloides* zygospore germination were not successful with the conditions and isolates tested.

Mating ultrastructures were observed by scanning electron microscopy analysis. Mating between strains ATCC1216a(−) and ATCC1216b(+), and also vegetatively grown ATCC1216b cells, were examined to investigate sexual and asexual morphologies. Zygospores are morphologically distinct from asexual spore-harboring structures, sporangia, which develop at the apex of aerial hyphae (Figure 8B). The zygospores were thick-walled and enveloped by repeated asterisk-like structures. Zygospores are the dormant, stress-tolerant stage, and thus these structures may contribute to the increased rigidity of the sexual spores. *M. circinelloides* formed coiled hyphae, possibly during the process of conjugation of two mating type hyphae. These early stages of sexual development resemble mating structures in some dimorphic ascomycetes including *Histoplasma capsulatum* [41] and the dermatophyte *Microsporum gypseum* [42] (Figure 8C). A related zygomycete, *P. blakesleeanus*, forms a twisted rope-like structure prior to zygophore formation [23], whereas in *M. circinelloides* it is speculated that the formation of coiled hyphae is followed by hyphal fusion between the two mating types, and then by zygophore and mature zygospore formation.

### *sex* locus in the *Mucor circinelloides* subspecies

**Figure 9:**
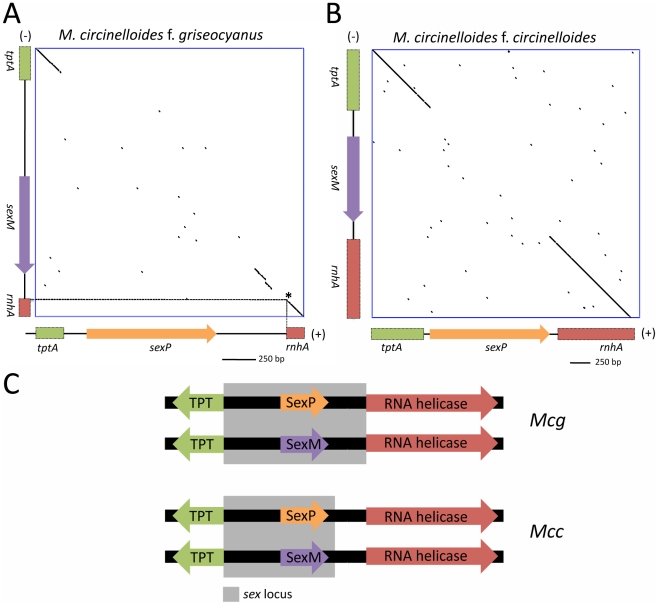
Defining the (+) and (−) *sex* locus alleles in *M. circinelloides* f. *griseocyanus* and *Mucor circinelloides* f. *circinelloides*. (A) Dot plot comparison of the *sex* locus alleles of the (+) and (−) strains of *Mcg* and *Mcc* show the sex specific regions with conserved flanking gene regions. The *tptA* and *rnhA* genes encoding the TPT and RNA helicase, respectively, are highly conserved between the (+) and (−) alleles; however, the *sex* genes encoding HMG domain proteins are divergent in sequence. Interestingly, the promoters for the TPT and RNA helicase genes are part of the *sex* locus in *Mcg* (A) but only the TPT promoter is part of the *sex* locus in *Mcc* (B). The regions of the *tptA* and *rnhA* genes sequenced are depicted (5′ regions of the genes). *sex* loci of ATCC1207a (+) and ATCC1207b were sequenced for *Mcg* and those of NRRL3614(−) and NRRL3615 (+) were sequenced for *Mcc*. Dotted lines indicate the start of the *rnhA* gene. Asterisk indicates the start of the RNA helicase gene. (C) The promoters for the TPT and RNA helicase genes are part of the *sex* locus in *Mcg* but only the TPT promoter is part of the *sex* locus in *Mcc*.

In previous studies, the *sex* locus of *P. blakesleeanus* was defined and found to contain one of two divergent HMG domain genes, *sexM* or *sexP* [23]. The *P. blakesleeanus sex* locus was compared with that of *M. c.* f. *lusitanicus* (*Mcl*) [22]. The synteny of the TPT-HMG-RNA helicase genes was found to be conserved in the two other subspecies of *M. circinelloides*, including *Mcg* and *Mcc* (Figure 9). Two *Mcg* strains, ATCC1207a (+) and ATCC1207b (−), were analyzed using primers (Table S2) designed from the genes for the triose phosphate transporter (*tptA*) and RNA helicase genes (*rnhA*) of the sequenced *Mcl* strain. The architecture of the *Mcg sex* locus, including gene order and orientation, was identical to that of *Mcl* (Figure S6), whereas the orientation of the *sexP* gene in *P. blakesleeanus* is opposite to that in the three *M. circinelloides* subspecies. DNA dot plot analyses were performed to establish the boundaries of the *sex* locus of *Mcg* and *Mcc* (Figure 9A). A comparison with the *R. oryzae* (+) *sex* locus (∼9 kb) revealed that the *sex* locus of the three *M. circinelloides* species is substantially shorter [1552 bp for *Mcl* (+), 1579 bp for *Mcl* (−); 1573 bp for *Mcg* (+), 1811 bp for *Mcg* (−); 1495 for *Mcc* (+), 1673 bp for *Mcc* (−)] and does not contain a BTB/Ankyrin/RCC1 domain protein gene spanning approximately 4,000 bp in *R. oryzae*. Additionally, the *tptA* gene of *R. oryzae* is in the opposite orientation from the *tptA* gene of the *M. circinelloides* subspecies and *P. blakesleeanus* (Figure S6). The *R. oryzae* (−) *sex* locus allele also lacks the additional ORF that is found in the (+) *sex* locus [24], [26].

Sequence comparisons of the *sex* loci of the (+) and (−) mating types of the *M. circinelloides* subspecies are detailed in Tables 4 and 5. Although the overall architecture was similar, there was an interesting difference in the *sex* locus of the *Mcg* species, where the border of the *sex* locus includes the promoters of both the *tptA* and *rnhA* genes whereas the *sex* locus of *Mcl* includes only the *tptA* promoter (Figure 9C) [22]. In *P. blakesleeanus*, neither the *tptA* nor the *rnhA* gene promoters are part of the *sex* locus [23]. This is a strong indication of the plasticity of the *sex* locus involving expansion/contraction in the *M. circinelloides* subspecies complex (Figure S7).

**Table 4:** DNA and deduced protein sequence comparison between three subspecies.

		*Mcl* (−)			*Mcg* (−)			*Mcc* (−)		
		TPT	SexM	RNA helicase	TPT	SexM	RNA helicase	TPT	SexM	RNA helicase
*Mcl* (+)	TPT	99% (99%)			86%			84%		
	SexP		6% (14%)			9% (12%)			12% (11%)	
	RNA helicase			99% (99%)			85% (83%)			83% (88%)
*Mcg* (+)	TPT	87%			93%			94%		
	SexP		3% (11%)			4% (12%)			2% (7%)	
	RNA helicase			84% (88%)			95% (88%)			80% (84%)
*Mcc* (+)	TPT	83%			94%			98%		
	SexP		3% (6%)			3% (14%)			8% (13%)	
	RNA helicase			81%			71%			98%

The lower scores of the TPT and RNA helicase similarity (93% and 95%, respectively) in *Mcg* (99% in *Mcl* and 98% *Mcc*) may be correlated with expansion of *sex* locus in *Mcg* or more recent gene conversion flanking the *sex* locus in *Mcl* and *Mcc*.

Numbers in the parentheses are for protein alignment scores.

In cases of differences in size between loci, sequences were trimmed for alignment.

**Table 5:** Comparison of SexP (left) and SexM (right) in the three subspecies.

		*Mcl* (+)	*Mcg* (+)			*Mcl* (−)	*Mcg* (−)
		SexP				SexM	
*Mcl* (+)	SexP	100% (100%)	85% (81%)	*Mcl* (−)	SexM	100% (100%)	85% (77%)
*Mcg* (+)	SexP	85% (81%)	100% (100%)	*Mcg* (−)	SexM	85% (77%)	100% (100%)
*Mcc* (+)	SexP	78% (74%)	78% (71%)	*Mcc* (−)	SexM	73% (54%)	73% (58%)

Numbers in the parentheses are for protein alignment scores.

## Discussion

### Virulence of the *M. circinelloides* subspecies and correlations between virulence, spore size, and the *sex* locus

*Mcl*, especially (−) mating type, was found to be highly virulent in the wax moth host. The difference in virulence between closely related species is of interest. One important difference between the mating types is spore size, in which only the (−) mating type of *Mcl* is highly virulent and produces larger spores. In the pathogenic basidiomycete *Cryptococcus neoformans*, the *MAT* locus is linked to virulence [43]; α mating type isolates are more prevalent in clinical isolates, the α *MAT* locus genes are highly expressed during infection in macrophages [44], and α isolates are more pathogenic in certain strain backgrounds [45], [46] or during co-infection [44], [47], [48]. The *sex* locus might therefore be similarly involved in the pathogenesis of this zygomycete species.

We found evidence that in *M. circinelloides*, the *sex* locus may be involved in virulence via a role in the asexual spore size of different mating types. These results prompted us to consider three models for the relationship between the *sex* locus and spore size. First, the *sex* locus could control spore size. In this model, SexM could have been required for larger spore size; however, the large spore size of the *sexMΔ* mutants isolated and characterized here exclude this model (Figure 5). Alternatively, SexP may promote smaller spore size, and this can be addressed by constructing isogenic mating type strains in which *sexM* is replaced with *sexP* or in which SexP has been deleted. Second, the *sex* locus and other unlinked genomic loci may together control spore size. In this model, spore size is a quantitative trait, and the *sex* locus may be one of several genes that contribute to control spore size. The (−) mating type isolate NRRL1443 has an intermediate spore size, possibly lending support to this hypothesis. In this model, deletion of *sexM* or *sexP* could lead to an intermediate spore size, possibly dependent on strain backgrounds, rather than strictly large or small spores. Third, the *sex* locus could play no role in controlling spore size. In this model, the apparent linkage observed between mating type and spore size could be the result of analysis of a small sample size. And it may not be the case that the *sex* locus contributes to virulence in ways other than spore size because the *sexMΔ* mutants are as virulent as wild-type (Figure 5). For example, the larger spore isolates could represent naturally occurring mutants that bypass a hypothetical cell cycle inhibition stage during spore dormancy and the multinucleate sporangiospores of larger size may reflect uncontrolled cell cycle: inside sporangia the spores would therefore break dormancy and undergo rounds of nuclear division. In this case, activation or overexpression of cell cycle inhibitors may reduce spore size.

Why are larger spores more virulent? The short or absent isotropic growth period for larger spores, compared to the long phase observed prior to germ tube emergence for smaller spores, could be involved in the differences in virulence (Figure 2, Videos S1 and S2). The larger spores are likely poised to undergo rapid invasive hyphal growth compared to the smaller spores. The extended isotropic growth phase of the smaller spores would result in slow germ tube formation or a block or delay in germ tube emergence, and could reduce virulence in the host. Our studies on the response of macrophages to spores further supported this hypothesis, where larger spores engulfed by macrophages are still able to send germ tubes (Figure 3 and Videos S3, S4, and S5). This observation could reflect a recent study in zygomycosis that shows the germ tubes of *R. oryzae* cause more damage compared to spores in *in vitro* experiments with an umbilical vein cell line [14], [49]. Although we observed that both the larger and smaller *M. circinelloides* spores trigger cell death of cultured macrophages with prolonged incubation (data not shown), the invasive hyphal growth contributes to render larger spores more virulent than smaller spores. The macrophage cell death elicited by *Mucor* is also of considerable interest. A previous study showed that an aqueous *R. oryzae* extract can trigger apoptosis in cultured human leukemia cells [50]. Thus, *Mucor* spores may produce unknown components that trigger host immune cell death responses resulting in susceptibility.

The relationship of spore size to virulence is also a central question to emerge from our study. Although large spores are more virulent in *Galleria*, it is also possible that we would observe an opposite result in pulmonary infections in a murine inhalation model, because larger spores may be less likely to penetrate lung alveolar spaces compared to smaller spores. Comparing results between intranasal and intravenous routes of infection in the murine model will provide insight into whether the route of infection influences the relative virulence of larger and smaller spores following inhalation.

Recent findings on size dimorphism involving *Cryptococcus* are interesting to consider in light of our findings on *Mucor* spore size dimorphism. *C. neoformans* often forms significantly enlarged cells called giant or titan cells, which are known to be more virulent and less susceptible to the host immune system [51], [52]. Aspects of fungal cell gigantism differ between the two different pathogenic fungi: *C. neoformans* giant/titan cells are mononucleate and polyploid, but *Mucor* large spores are multinucleate. However, in both cases it is clear that enlarged fungal cells confer benefits to the fungal pathogens during host infection. The human pathogenic ascomycete *Coccidioides immitis* is also known to exhibit cell giantism during host infection, where ‘smaller’ athroconidia undergo multiple cell cycles resulting in the formation of enlarged multinucleate cells, spherules, which escape from host immune systems [53], [54]. Given these precedents, other examples of fungal size dimorphism linked to virulence likely remain to be discovered.

In the diabetic murine host system, *Mcc* displays higher virulence compared to *Mcl* and *Mcg* tested in this study (Figure 7 and Figure S5). This is an intriguing observation, which is in accord with the increased prevalence of the *Mcc* species in *Mucor* clinical isolates (Table 1 and Figure S4). *Mcl* isolates, especially larger spore isolates, are highly virulent in the wax moth host, however, they are less virulent in the murine host indicating that virulence traits may have been differentially adapted during speciation. For example, the *Mcl* isolates tested exhibited limited growth at 37°C but *Mcc* species are relatively resistant to the human/mouse body temperature (Figure 7 and data not shown). Interestingly not all *Mcc* strains show the same level of virulence (Figure S5), and further investigation is necessary to examine differences between virulent and less-virulent *Mcc* strains.

### Subspecies in the *M. circinelloides* complex

The *M. circinelloides* complex comprises three subspecies, *M. c.* f. *lusitanicus* (*Mcl*), *M. c.* f. *griseocyanus* (*Mcg*), and *M. c.* f. *circinelloides* (*Mcc*), and has been historically characterized based on physiological and morphological characteristics [31], or by comparing enzymes of certain *M. circinelloides* species [55]. Based on our MLST analysis, the *M. circinelloides* subspecies are closely related when compared to the *P. blakesleeanus* and *R. oryzae* outgroups, but there is enough sequence divergence in the *ITS*, *rDNA2*, and *RPB1* regions to support at least the current subspecies classifications (Figure 6). We found no examples of MLST marker exchange between the 3 subspecies and if more comprehensive analyses of the population substantiate this observation, they may represent cryptic species. Our mating assays showed mating occurs predominantly within the subspecies with only a few exceptional intersubspecies fertile combinations, further suggesting that they could represent cryptic species with genetic isolation limiting or preventing introgression. Phylogenic analysis with SexP and SexM indicates that allelic sex determinant genes may have evolved before speciation within zygomycetes, especially in the Mucorales (Figure S8). Allele compatibility tests support evidence for recombination in the clinical *Mcc* population (Figure S9).

### The *sex* locus governs sexual reproduction of the *M. circinelloides* subspecies

Several criteria have been used to define the *sex* locus in the heterothallic zygomycetes [22], [23], [26]. First, the (+) and (−) mating types are defined by the presence of the *sexP* or *sexM* genes, respectively. Mating is only observed between opposite mating types. Additionally, rare *Phycomyces* disomic strains containing both *sexP* and *sexM* are self-fertile, producing spiral, zygospore-like structures [23]. Furthermore, the *sex* locus region has been genetically mapped with crosses and RFLP analysis, linking the *Phycomyces sexP* gene to the (+) mating type and likewise, the *Phycomyces sexM* gene to the (−) mating type within a 38 kb interval linked to mating type. Most importantly, the *Mucor sexMΔ* mutants isolated in this study are sterile (Figure 6). Finally, the sex-determining region has been corroborated across *R. oryzae*, *M. circinelloides*, and *P. blakesleeanus*, representing three species within the Mucorales.

Our SEM analyses of the early development of zygospores revealed a coiled hyphae structure before the rest of the zygophore is produced (Figure 4). Formation of coiled hypha was previously described in *H. capsulatum*, in which the hyphae of one mating type extends and enwraps the hyphae of the opposite mating type [56]. Branches form around this knob-like structure and anastomoses between the hyphae result in a larger hyphal mass that eventually becomes the ascocarp. It is possible that zygospore formation in *M. circinelloides* could follow a similar process of hyphal mass aggregation, followed by anastomoses culminating in formation of the zygospore. However, the factors that contribute to the remarkable rigidity of the zygospore have yet to be discovered.

Interestingly, the promoter of the TPT gene is included within the *sex* locus for *Mcl* while the promoter lies outside the *sex* locus of *P. blakesleeanus* [26]. In this study, we found that in addition to the TPT gene, the promoter for the RNA helicase gene is also included in the *sex* locus for *Mcg*. This is indicative of a possible expansion, or contraction, of the *sex* locus in the different subspecies by changing the recombination block that punctuates the evolutionary trajectory of this dynamic region of the genome (Figure S6). Evolutionarily, the TPT and RNA helicase region may have been included or excluded from the *sex* locus over time [26]. Thus, our observations may imply an expansion or contraction of the *sex* locus in zygomycetes, especially in the *M. circinelloides* complex (Figure S7). *MAT* locus expansion/contraction has been observed in ascomycetes. The *MAT* locus of ascomycetes is generally characterized as a syntenic region with *APN1-MAT1-1* (alpha box)-*SLA2* or *APN1-MAT1-2* (HMG)-*SLA2* gene clusters [57], [58]. In two evolutionarily related ascomycetous fungal groups, the dermatophytes and dimorphic fungi, the *APN1*, *MAT1-1* or *MAT1-2*, and *SLA2* genes span ∼3 kb in *Microsporum gypseum* compared to ∼9 kb in *Coccidioides immitis/posadasii* in which flanking genes have been recruited into the *MAT* locus [42].

Comparison of *sex* and *sex*-related loci in zygomycetes and microsporidia also revealed additional ORFs in the *sex*/*sex*-related locus [59]. This might suggest gene eviction from or capture into the *sex* locus. Examples can be found in pathogenic basidiomycete tetrapolar *MAT* loci. The basidiomycete tetrapolar *MAT* locus involves a homeodomain locus and a pheromone/receptor locus. In *Ustilago maydis*, a plant pathogenic basidiomycete, the *MAT* locus harbors unlinked homeodomain and pheromone/receptor loci and the *MAT* locus spans ∼4 kb for the homeodomain and ∼4.4–8 kb for the pheromone/receptor locus. In *C. neoformans* the two domains are linked and novel genes have been captured into a bipolar *MAT* locus that now spans >100 kb [26]. The bipolar *MAT* loci of *U. hordei* and *Malassezia globosa* are even larger and span more ∼500 kb or ∼170 kb, respectively [60]–[62]. The *MAT* loci of *C. neoformans*, *U. hordei*, and *M. globosa* serve as dramatic examples of *MAT* locus expansion and share features with sex chromosome evolution in plants and animals. Additionally, a comparison of the *MAT* locus within the *Cryptococcus* complex suggests an evolutionary trajectory involving gene evictions [63], [64]. In *C. neoformans* var. *grubii* and *C. gattii*, the *MAT* locus includes three genes (*IKS1*, *NCM1*, and *BSP3*), although in *C. neoformans* var. *neoformans* these three genes have been evicted from the *MAT* locus by gene conversion leading to a relative contraction of the *MAT* locus. Similar molecular events may have punctuated the evolution of the zygomycete *sex* locus.

## Materials and Methods

### Ethics statement

The animal studies at Duke University Medical Center were in full compliance with all of the guidelines of the Duke University Medical Center Institutional Animal Care and Use Committee (IACUC) and in full compliance with the United States Animal Welfare Act (Public Law 98–198). The Duke University Medical Center IACUC approved all of the vertebrate studies. The studies were conducted in the Division of Laboratory Animal Resources (DLAR) facilities that are accredited by the Association for Assessment and Accreditation of Laboratory Animal Care (AAALAC).

### Strains and media

The strains used in this study are listed in Table 1 and in Table S1. *M. circinelloides* strains were grown on yeast and dextrose agar (YPD) or potato dextrose agar (PDA) media for spore production at room temperature. For mating, YXT (4.0 g yeast extract; 10 g malt extract; 4 g glucose; 15 g agar; 1000 mL water, with the pH adjusted to 6.5) [65], YPD (10 g yeast extract; 20 g peptone; 20 g dextrose; 20 g agar; 1000 mL water), and V8 media (50 mL V8 juice; 0.5 g KH_2_PO_4_; 950 mL dH_2_O; 40 g bactoagar at pH between 7.0–7.2 adjusted with 5 M KOH) were used. Plasmids in this study were maintained in *Escherichia coli* One Shot MAX Efficiency DH5α-T1R competent cells (Invitrogen Co., Carlsbad, CA) and manipulated as previously described [66]. Microbial strains were grown under appropriate Biosafety Level 2 conditions (BSL2). All chemicals for media, buffer, and supplements were from Difco Laboratories (Detroit, MI) unless otherwise indicated.

According to the Centraalbureau voor Schimmelcultures (CBS), American Type Culture Collection (ATCC), and ARS Culture Collection (NRRL) databases, the strains CBS277.49, ATCC1216b, and NRRL3631 are the same isolate with different designations. However, previous studies indicate that these isolates differ in karyotype [67], [68]. Our further analysis demonstrated the three isolates including another ATCC isolate, ATCC1216a, have different distinguishing SNPs in the *RPB1* gene. The database mating type designation for these isolates are inconsistent with our data; we find that CBS277.49 and ATCC1216a are (−); NRRL3631 and ATCC1216b are (+). CBS277.49, NRRL3631, and ATCC1216a are different based on analyses by Random Amplification of Polymorphic DNA (RAPD). These results document that while those isolates are all clearly isolates of *Mcl* they are genetically distinct, despite the records of the stock culture collections (See Supplementary Text S1).

### Virulence assays

Spores were resuspended in a phosphate buffered saline (PBS). PBS containing 500 or 1,000 spores or 5 µl of PBS alone were injected into wax moth (*Galleria mellonella*) larvae (10 or 20 larvae per strain). For the murine host model, groups of BALB/c mice were rendered diabetic with 190 mg per body kg streptozocin (in citric acid buffer pH = 4.5) through intraperitoneal injection 10 days prior to fungal challenge [14]. A cohort of injected mice (10) was randomly chosen and confirmed to exhibit glycosuria with Keto-Diastix reagent strips (Bayer Co. Elkhart, IN). After 10 days, the mice were infected with 10^6^ spores in 200 µl PBS through tail vein injection. Survival rate of the host was monitored twice a day and body weight was measured daily. Animals that appeared moribund or in pain were sacrificed appropriately. Significance of mortality rate data was evaluated by using Kaplan–Meier survival curves with the PRISM statistics software (GraphPad Software, Inc., La Jolla, CA).

### Light, scanning electron, and transmission electron microscopy

Spores were observed with a Zeiss Axioskop 2 Plus with an AxioCam MRm camera (Carl Zeiss Inc., Thornwood, NY). To analyze nuclei, spores were fixed with 3.7% formaldehyde in 50 mM potassium phosphate buffer, pH 7.0, containing 0.2% Triton X-100. Then the spores were mounted on a coverslip with ProLong Gold antifade reagent with DAPI (Invitrogen, Carlsbad, CA.). For confocal microscopic analyses, a Zeiss LSM 510 inverted confocal microscope was used (Carl Zeiss Inc., Thornwood, NY). Time lapse analyses for large and small spore germination, and interactions between macrophages and spores were performed by using the Zeiss Axio Observer Z1 microscope system (Carl Zeiss Inc., Thornwood, NY) equipped with an Opto-electonically motorized XY stage, Pecon XL S1 incubator, and Coolsnap ES2 high resolution CCD camera (Photometrics Inc., Huntington Beach, CA). A 6-well plate was layered with YPD media and inoculated with spores of R7B and NRRL3631. For macrophages, J774 murine macrophage cell lines were layered on a 6-well plate at 10^5^ cells/ml prior to the fungal spore challenge. The same number of spores (10^5^ spores/ml) was inoculated into 6-well plates and the plates were immediately observed by microscopy. The images were obtained every 30 seconds and reconstructed as a movie by using MetaMorph 7.6.5 (Molecular Devices Inc., Sunnyvale, CA).

For scanning electron microscopy (SEM), the mating/culture plates were washed with 0.1 M Na cacodylate buffer (pH = 6.8), and 1 mm^3^ blocks of mating areas were excised, and incubated in fixation buffer at 4°C. Samples were then rinsed in cold 0.1 M Na cacodylate buffer three times, post-fixed in 2% osmium tetroxide in 0.1 M Na cacodylate buffer for 2.5 h at 4°C, critical point dried, and sputter coated before being viewed by SEM.

Additional SEM of *M. circinelloides* spores was accomplished as follows. The R7B and NRRL3631 strains were inoculated on PDA and spores were collected after 4 days. Spores were suspended in 0.1 M sodium cacodylate and immobilized on Millipore Nitrocellulose filters (Millipore HAWP 0.46 µm). The spores and membrane were immediately fixed in 2% glutaraldehyde (Electron Microscopy Sciences, EMS, Hatfield, PA), 0.05% malachite green oxalate (EMS) in 0.1 M sodium cacodylate buffer, and incubated at 4°C until further processing. The fixation buffer was then removed and the membrane was washed in 0.1 M sodium cacodylate prior to being subjected to an ethanol dehydration series (2 times for 10–15 min in 25%, 50%, 75%, 95%, and 3 times in 100% ethanol). Samples were then critical point dried (Pelco CPD2, Ted Pella, Inc., Redding, CA), sputter coated, and viewed and imaged with the FEI XL30 SEM-FEG (FEI Company, Hillsboro, OR) at the Shared Materials Instrument Facility (SMIF) at Duke University.

Transmission electron microscopy (TEM) of *M. circinelloides* spores was accomplished as follows. Spores were collected as described above and washed in 0.1 M sodium cacodylate buffer (pH = 6.8), collected by centrifugation (∼4,000 rpm, 3 min in a table top centrifuge), resuspended in 2% glutaraldehyde plus 0.05% malachite green oxalate in 0.1 M sodium cacodylate buffer, and incubated at 4°C for 2 days. Fixed spores were collected by centrifugation, resuspended, washed once with 0.1 M sodium cacodylate buffer, centrifuged, the supernatant was removed, and 100–200 µl 1.6% agarose was added to the tube on ice to immobilize the cells in a 0.8% agarose pellet. The agarose pellet containing spores was then dehydrated by an ethanol series (2 times for 10–15 min in 25%, 50%, 75%, 95%, and 3 times in 100% ethanol), and then stained with 0.8% K_3_Fe(CN)_6_, 1% OsO_4_, 0.1 M sodium cacodylate for 1 hr at room temperature. The agar pellet was then washed two times with 0.1 M sodium cacodylate buffer and stained with 1% tannic acid for 1 hr at room temperature. The pellet was then washed with 0.1% sodium cacodylate buffer for 5 min followed by two washes in ddH_2_0 for 5–10 min each, and then stained with 1% uranyl acetate in water overnight at 4°C. Sample were then prepared for embedding in Embed812 (EMS) as follows, one 5 min incubation in 50/50 ethanol-propylene oxide, three 10 min incubations at room temperature in 100% propylene oxide, 50/50 Embed812-propylene oxide overnight at room temperature with gentle rotation, 10 min in 100% uncatalyzed Embed812, and 1 hr in 100% catalyzed Embed812. Catalyzed Embed812 was then drained off, agar pellets are immersed in 100% catalyzed Embed812 beam capsule, and cured at 65°C for 72–96 hrs. The cured peg was then trimmed, sectioned, and mounted on copper grids. Grids were post-stained prior to viewing. Sections were viewed and imaged with a Philips/FEI CM 12 Transmission EM instrument (FEI Company, Hillsboro, OR) with Advanced Microscopy Techniques, Corp. (AMT) 2k×2k digital camera (Danvers), Duke University Department of Pathology or by the FEI Tecnai G^2^ Twin instrument at the Shared Materials Instrument Facility (SMIF) at Duke University.

### Disruption of the *sexM* gene

To disrupt the *sexM* gene in the *sex* locus, we constructed a disruption allele containing the *pyrG* gene flanked by 1 kb each of the 5′ and 3′ regions of the *sexM* gene by using overlap PCR. The 5′ end was amplified with primers JOHE20368 and JOHE20369 and the 3′ end was amplified with primers JOHE20372 and JOHE20373 (Table S2). The *pyrG* fragment was amplified with primers JOHE20370 and JOHE20371 from the genome of wild type *Mcl* strain CBS277.49. The three fragments were then subjected to an overlap PCR to isolate a disruption allele as described [69]. The cassette was purified and strain MU402 (*leuA^−^*, *pyrG^−^*) was transformed to disrupt the target gene and transformation was carried out essentially as described previously [70]. In brief, protoplasts were obtained from 2.5×10^8^ germinated spores of strain MU402 *(pyrG^−^*, *leuA^−^*) [34] by incubation with 0.03 unit/ml chitosanase RD (US Biologicals) and 1 mg/ml Lysing Enzymes (L-1412; Sigma) at 30°C for ∼90 min. Protoplasts were incubated with 50 µg DNA and *pyrG* ^+^ transformants were selected in MMC medium (1% casamino acids, 0.05% yeast nitrogen base without amino acids and ammonium sulfate, 2% glucose) pH 3.2, supplemented with 0.5 M sorbitol [34]. Because the initial transformants are usually heterokaryons due to the presence of several nuclei in the protoplasts, transformants were grown in MMC selective medium for several vegetative cycles to increase the proportion of transformed nuclei.

Two *pyrG*-positive transformants were finally selected and homologous replacement was confirmed by PCR and Southern blotting. In brief, primers; P1 and P2, were used to identify the integration of the *pyrG* gene at the *sexM* locus. The genomic DNA of the disruption candidates and wild-type were digested with SalI and Southern blotting was performed with the 3′ fragment as a probe as described [66]. *M. circinelloides* hypha is coenocytic, therefore a vegetative passage is required to select progeny only with transformed nuclei. During this step, the two initial transformants contained different proportions of transformed nuclei and we interpret these to be the result of independent transformation events though they were isolated from the same transformation experiments.

### Sequencing, assembly, and bioinformatics

Primers used in this study are listed in Table S2. Primers JOHE19916 and JOHE19917 were used to amplify the entire *MAT* locus for all three subspecies of *M. circinelloides*. All other primers were used for subsequent PCR analysis and sequencing. Primers JOHE19868 and JOHE19869 were used to amplify the TPT gene region and JOHE22785, JOHE22786, JOHE22787, JOHE22788, JOHE22789, and JOHE22790 were used to amplify the RNA helicase gene region.

For sequencing of the *sex* locus and MLST analysis of *M. circinelloides* strains, PCR products were cloned into plasmid pCR2.1-TOPO following the manufacturer's instructions (Invitrogen, Carlsbad, CA) or subjected to direct sequencing of the PCR product. Sequencing reactions were performed with an Eppendorf epgradient thermal cycler using standard BigDye Terminator chemistry (Applied Biosystems, Foster City, CA) and sequencing was carried out at the IGSP sequencing facility at Duke University (http://www.genome.duke.edu/cores/sequencing) using an Applied Biosystems 3730xl DNA Analyzer. DNA sequences were analyzed with Sequencher version 4.10 and BLAST [71]. Genes were annotated with FGENESH or ORF finder (NCBI). Obtained sequences were deposited in GenBank (Table S3).

### Phylogenetic analysis

Sequences were analyzed with CLUSTALW and phylogenies were constructed using a PhyML 3.0 software [72], which allowed phylogenies to be inferred and levels of support ascertained, and PAUP 4.0 (Sunderland, MA). A species tree was constructed by concatenating two conserved loci, *rDNA2* and *RPB1*, from the fungal tree of life project [2]. Phylogenetic trees were drawn with the Dendroscope program [73] with aligned sequences.

### Mating assays

All mating assays were performed on YPD, YXT, or V8 media. Fungal strains were grown on PDA for 2 days, and two agar blocks (5 mm×5 mm) containing mycelia of each strain were then placed approximately 1 cm apart at the edge of a mating plate. All possible combinations of strains listed in Table 2 were tested. Plates were incubated for 3 days at 15°C and then for another 4 days at room temperature. All mating plates were incubated in the dark. Zygospore and mating specific structure formation were monitored with a Nikon Eclipse E400 microscope, equipped with a Nikon DXM1200F digital camera (Nikon Instrument Inc., Melville, NY).

## Supporting Information

Figure S1 Mating type, spore size, and virulence in *M. circinelloides* f. *lusitanicus*. (A) (+) mating type isolates produce smaller spores, whereas three (−) isolates including CBS277.49, ATCC1216a, and CBS108.17 produce significantly larger spores (p<0.0001) and 7 (−) isolates produce intermediate sized spores. One hundred spores of each isolate were examined. Y axis is the size of spores (µm). (B) The larger spores are more virulent in the wax moth host model (P = 0.0002 in CBS108.17 vs. CBS969.68). These results indicate that there is a possible correlation between spore size and mating type and that spore size is a virulence factor.
(TIF)

Figure S2 Sporangia of (−) and (+) strains of *M. circinelloides* f. *lusitanicus*. Strain R7B(−) are large inside the sporangia (upper), whereas strain NRRL3631 (+) produces homogeneously small spores (bottom). Scale = 40 µm.
(TIF)

Figure S3 PCR confirmation of disruption of the *sexM* gene and mating assays of the *sexMΔ* mutants. (A) Primers downstream of the *sexM* (P1) and *pyrG* gene (P2) amplified an ∼1.7 kb PCR product that was expected when the *sexM* gene was replaced with the *pyrG* gene (B). Another primer that is outside of the disruption cassette, 5′ upstream of *sexM* (JOHE19868) produced a 2,122 bp wild-type specific PCR product with a primer (JOHE19784) within the *sexM* gene or 3,382 bp mutation specific PCR product with the primer (JOHE20371) to the *pyrG* gene (C). Both *sexMΔ* mutants are unable to mate with either (−) or (+) mating type strains. Arrows indicate a dark line of zygospores indicative of mating of fertile isolates (D).
(TIF)

Figure S4 Phylogenetic analyses by MLST analysis of the clinical isolates using the *RBP1*, *rDNA2*, and *ITS* loci. As shown, most clinical isolates cluster with the previously defined *Mcc* subspecies. Interestingly, we found evidence for a fourth group containing ATCC1209b and UIC-1 in all three trees. All trees were constructed with 50 bootstrap replicates. Red indicates clinical isolates.
(TIF)

Figure S5 Virulence of clinical *Mucor* isolates in the diabetic murine host model. Six *M. circinelloides* f. *circinelloides* strains display significantly higher virulence (for example, *p* = 0.0021 for CNRMA03.154 vs. D-tate indicates the curves are significantly different), whereas three non-*Mcc* isolates are avirulent under these conditions. Two *Mcc* isolates, NRRL3615 and CBS195.68, also exhibit reduced virulence compared to the other *Mcc* isolates. The black curve represents NRRL3631, D-tate, AS71, and UIC-1.
(TIF)

Figure S6 The genomic organization of the zygomycete *sex* locus. The *sex* locus alleles of *P. blakesleeanus*, *M. c.* f. *lusitanicus*, *M. c.* f. *griseocyanus*, *M. c.* f. *circinelloides*, and *R. oryzae* are shown. The *sex* locus is in grey. The *tptA* genes of *Mcg* and *Mcc* and the *rnhA* gene of *Mcc* (+) were not fully sequenced and are depicted in white with dotted outlines assuming conservation among the *M. circinelloides* subspecies. *M.c.* = *Mucor circinelloides*.
(TIF)

Figure S7 Hypothetical evolutionary trajectory of the *sex* locus. In the *M. circinelloides* complex, the *sex* locus could either expand (A) or contract (B) in size incorporating or evicting bordering genes or gene promoters as observed in other fungi (see text for details). Gene sizes are not to scale.
(TIF)

Figure S8 Unrooted phylogenetic tree for the SexP and SexM proteins. SexP proteins from different zygomycetes form a cluster, as do the SexM proteins, indicating that allelic sex determinant genes may have evolved before speciation within zygomycetes, especially in the Mucorales. Human HMG proteins (Sry, Sox3, Sox5, Sox11, and Sox13) were used as an outgroup. Pb: *P. blakesleeanus*, Ro: *R. oryzae*, Mcc: *M. circinelloides* f. *circinelloides*, Mcg: *M. circinelloides* f. *griseocyanus*, and Mcl: *M. circinelloides* f. *lusitanicus*. Scale = 0.5 (base pair substitution per base).
(TIF)

Figure S9 Possible sexual recombination within the clinical *M. circinelloides* isolates. Three loci, *ITS*, *rDNA2*, and *RPB1*, were examined to evaluate possible recombination within the *Mcc* species. Black lines indicate pairings of alleles within an isolate. Red lines highlight loci for which all four allele compatible combinations are observed (AB, ab, Ab, aB), providing evidence for recombination in the population.
(TIF)

Table S1 *M. circinelloides* f. *lusitanicus* strains used in this study.
(DOC)

Table S2 Primers used in this study.
(DOC)

Table S3 GenBank accession numbers for *ITS*, *rDNA2*, *RPB1*, and *sex* locus.
(DOC)

Text S1 Strain typing with respect to CBS277.49, ATCC1216b, ATCC1216a, and NRRL3631.
(DOC)

Video S1 Germination of larger spores. The larger spores display a shorter isotropic growth phase or bypass the isotropic growth stage resulting in a rapid and immediate germ tube emergence after exiting dormancy.
(MOV)

Video S2 Germination of smaller spores. The smaller (+) spores grow isotropically for a longer time until their size is comparable to that of the larger (−) spores, and they then start sending germ tubes.
(MOV)

Video S3 Interaction between larger spores (LS) and macrophages. The larger spores germinated inside of the macrophages.
(MOV)

Video S4 Interaction between smaller spores (SS) and macrophages. The smaller spores remained dormant inside macrophages without isotropic growth or germination, and grew significantly more slowly compared to the small spores outside of macrophages.
(MOV)

Video S5 Interaction between isotropically grown spores (IS) and macrophages. Interestingly, IS also germinated inside macrophages similar to the LS.
(MOV)
