# Supplementary figures and images for: Sporangiospore Size Dimorphism Is Linked to Virulence of Mucor circinelloides
Source: PLoS Pathog. 2011 Jun 16;7(6):e1002086. doi: 10.1371/journal.ppat.1002086 (PMC3116813; doi:10.1371/journal.ppat.1002086)

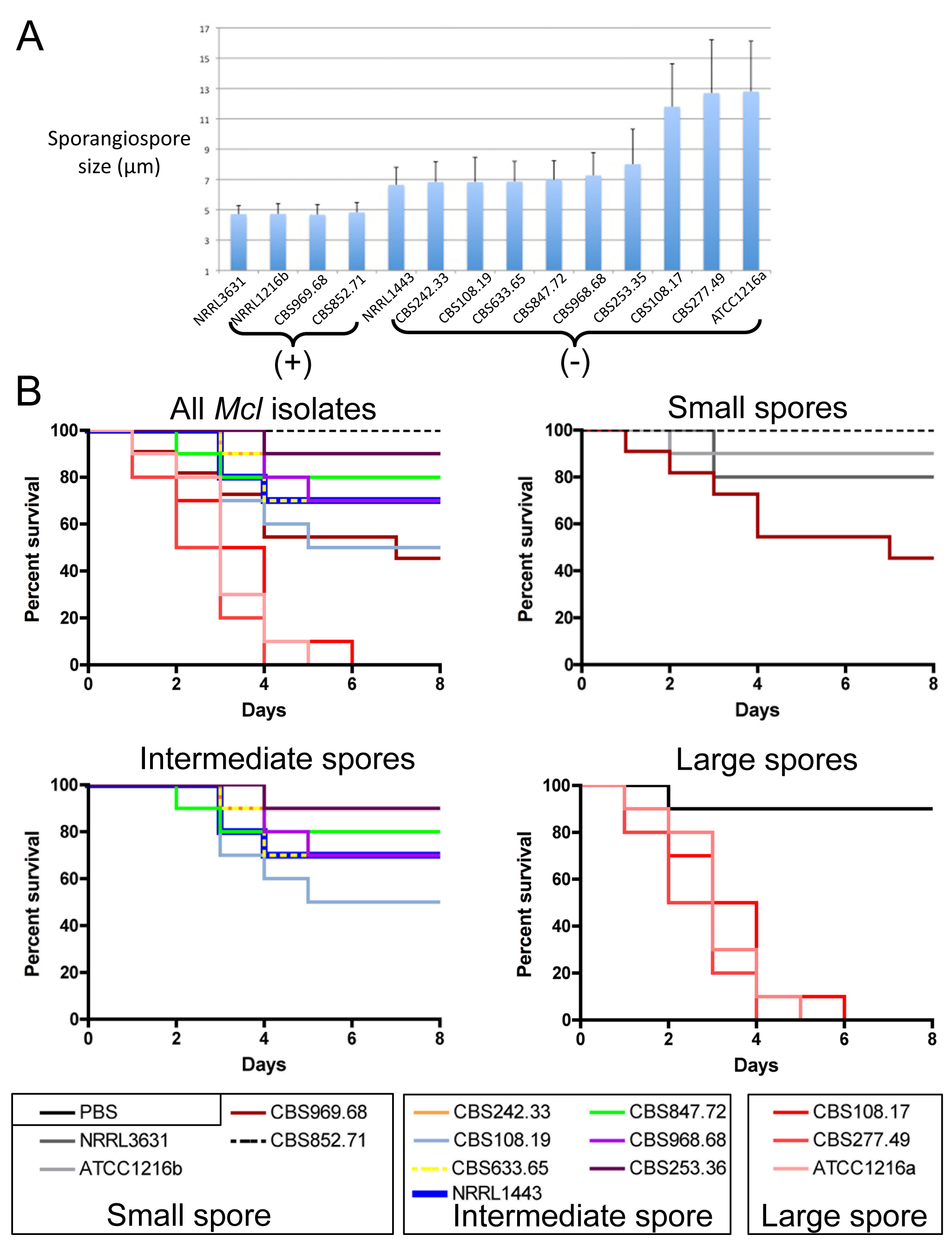

Supplement: Figure S1 — Mating type, spore size, and virulence in M. circinelloides f. lusitanicus. (A) (+) mating type isolates produce smaller spores, whereas three (−) isolates including CBS277.49, ATCC1216a, and CBS108.17 produce significantly larger spores (p<0.0001) and 7 (−) isolates produce intermediate sized spores. One hundred spores of each isolate were examined. Y axis is the size of spores (µm). (B) The larger spores are more virulent in the wax moth host model (P = 0.0002 in CBS108.17 vs. CBS969.68). These results indicate that there is a possible correlation between spore size and mating type and that spore size is a virulence factor. (TIF) [file ppat.1002086.s001.tif]

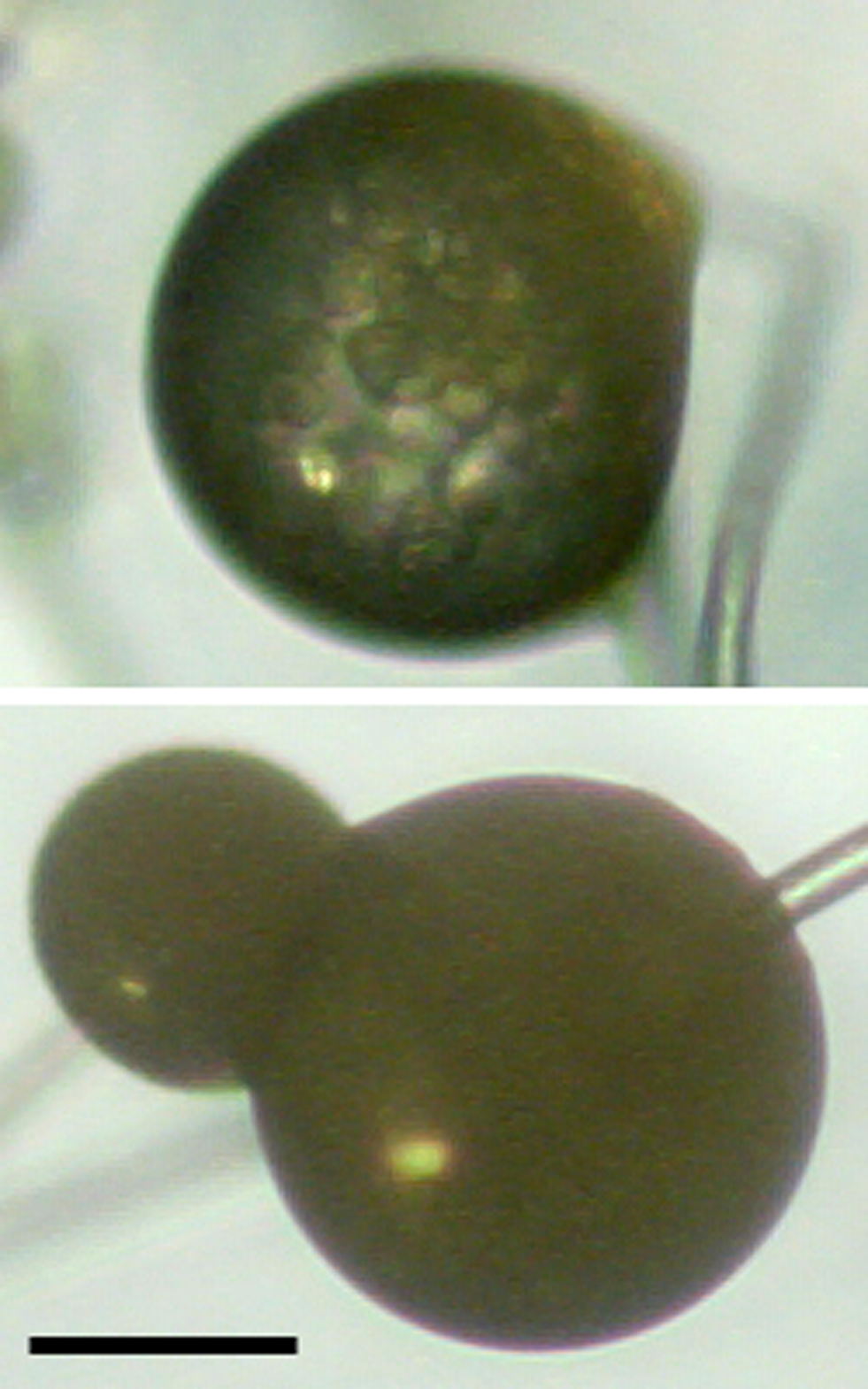

Supplement: Figure S2 — Sporangia of (−) and (+) strains of M. circinelloides f. lusitanicus. Strain R7B(−) are large inside the sporangia (upper), whereas strain NRRL3631 (+) produces homogeneously small spores (bottom). Scale = 40 µm. (TIF) [file ppat.1002086.s002.tif]

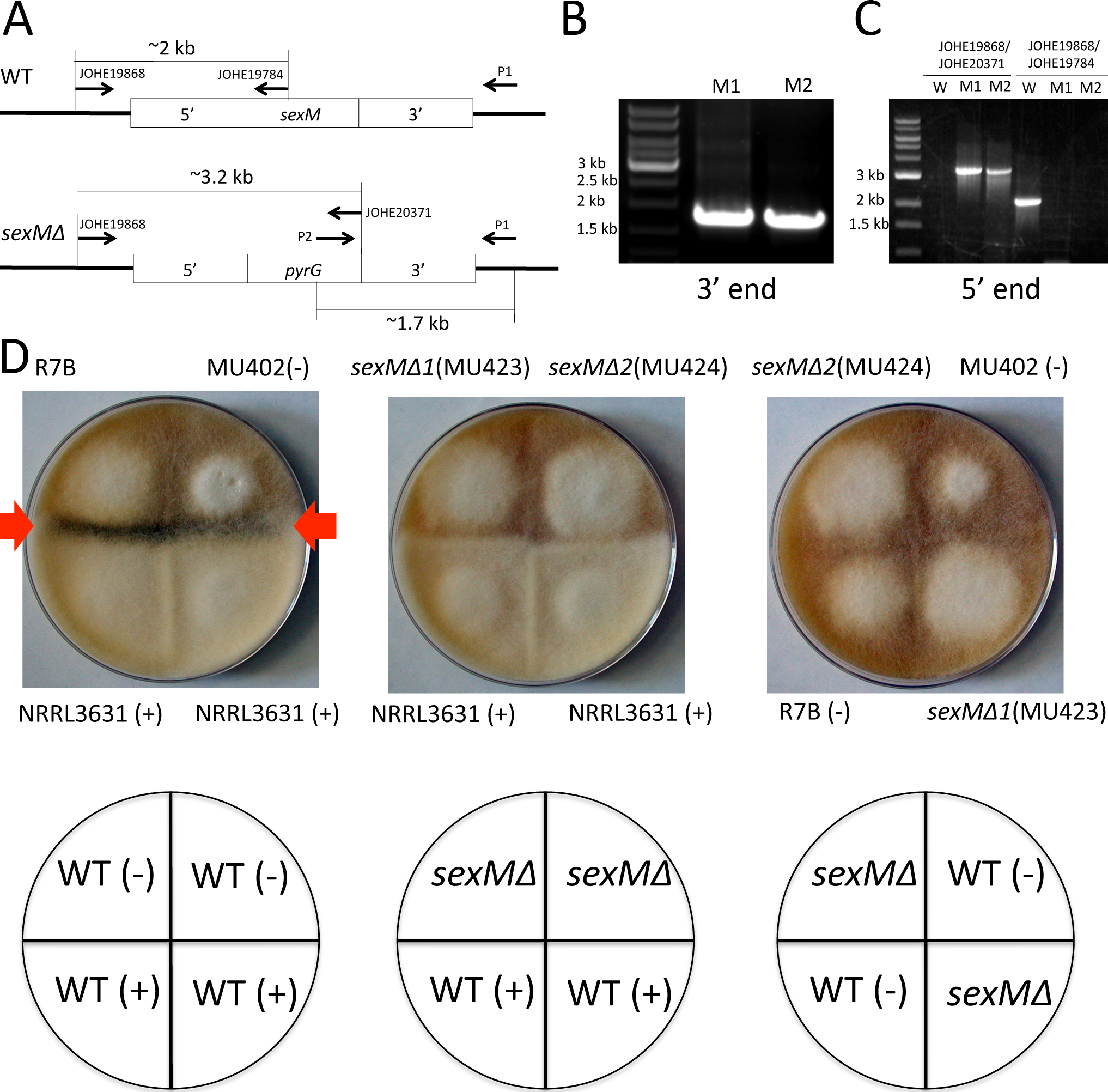

Supplement: Figure S3 — PCR confirmation of disruption of the sexM gene and mating assays of the sexMΔ mutants. (A) Primers downstream of the sexM (P1) and pyrG gene (P2) amplified an ∼1.7 kb PCR product that was expected when the sexM gene was replaced with the pyrG gene (B). Another primer that is outside of the disruption cassette, 5′ upstream of sexM (JOHE19868) produced a 2,122 bp wild-type specific PCR product with a primer (JOHE19784) within the sexM gene or 3,382 bp mutation specific PCR product with the primer (JOHE20371) to the pyrG gene (C). Both sexMΔ mutants are unable to mate with either (−) or (+) mating type strains. Arrows indicate a dark line of zygospores indicative of mating of fertile isolates (D). (TIF) [file ppat.1002086.s003.tif]

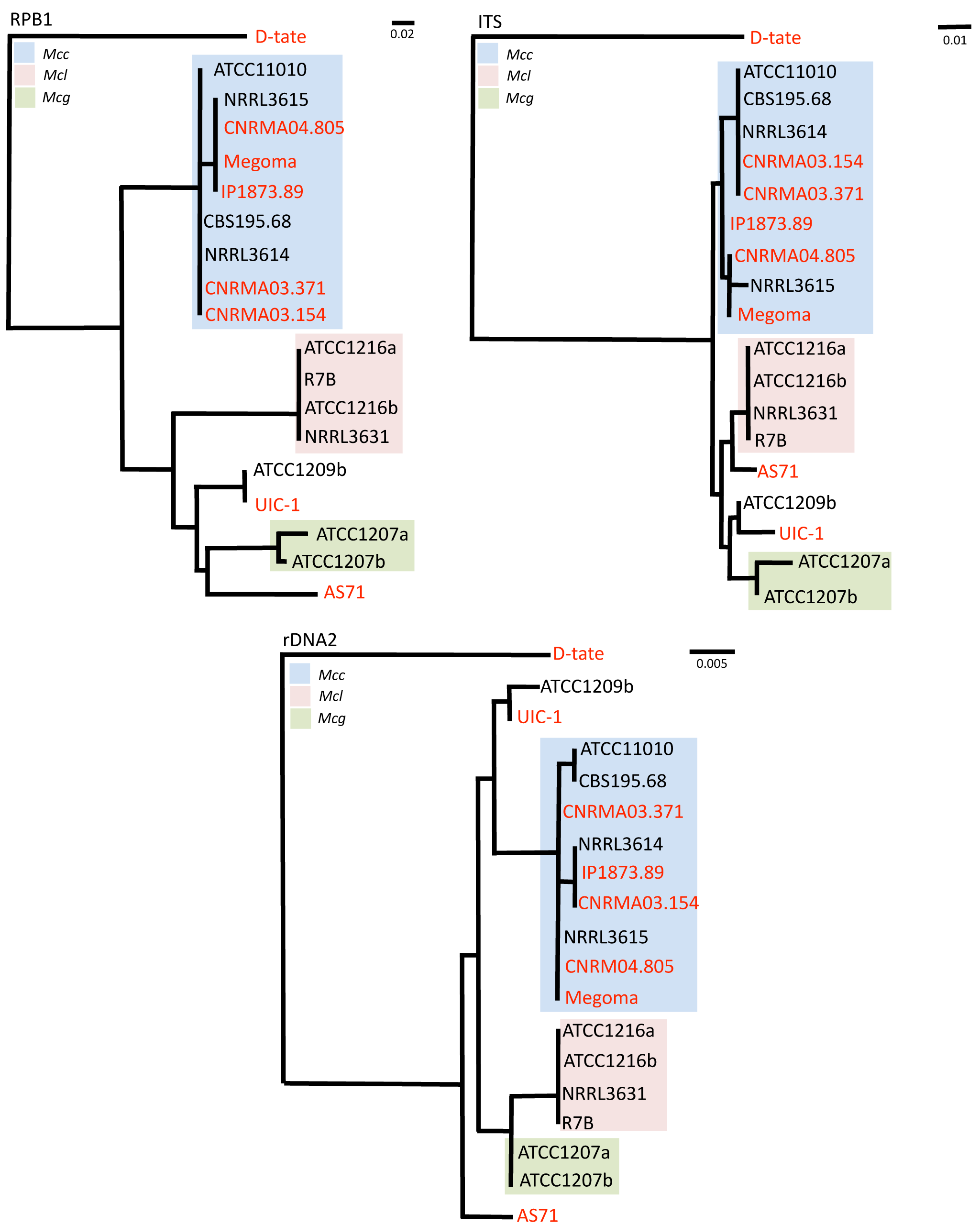

Supplement: Figure S4 — Phylogenetic analyses by MLST analysis of the clinical isolates using the RBP1, rDNA2, and ITS loci. As shown, most clinical isolates cluster with the previously defined Mcc subspecies. Interestingly, we found evidence for a fourth group containing ATCC1209b and UIC-1 in all three trees. All trees were constructed with 50 bootstrap replicates. Red indicates clinical isolates. (TIF) [file ppat.1002086.s004.tif]

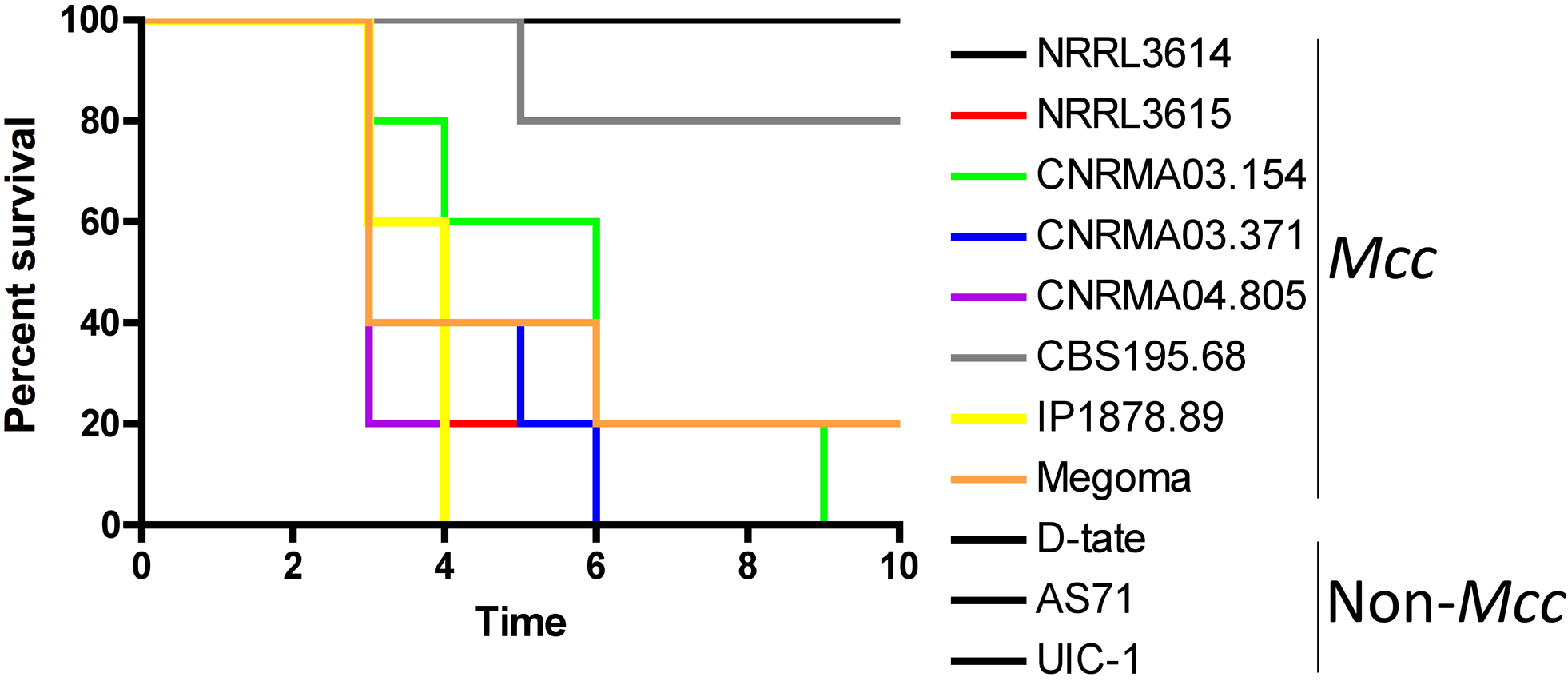

Supplement: Figure S5 — Virulence of clinical Mucor isolates in the diabetic murine host model. Six M. circinelloides f. circinelloides strains display significantly higher virulence (for example, p = 0.0021 for CNRMA03.154 vs. D-tate indicates the curves are significantly different), whereas three non-Mcc isolates are avirulent under these conditions. Two Mcc isolates, NRRL3615 and CBS195.68, also exhibit reduced virulence compared to the other Mcc isolates. The black curve represents NRRL3631, D-tate, AS71, and UIC-1. (TIF) [file ppat.1002086.s005.tif]

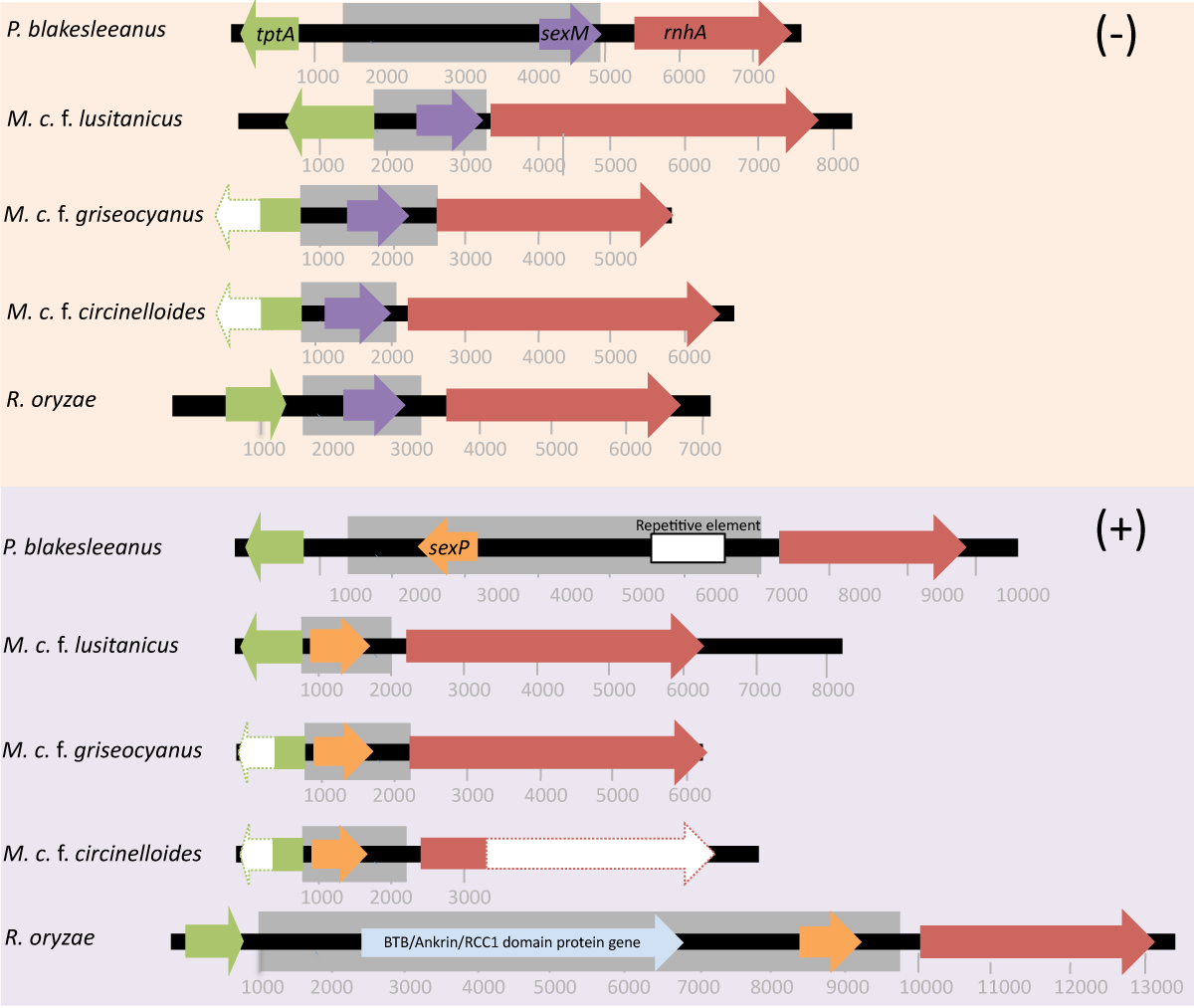

Supplement: Figure S6 — The genomic organization of the zygomycete sex locus. The sex locus alleles of P. blakesleeanus, M. c. f. lusitanicus, M. c. f. griseocyanus, M. c. f. circinelloides, and R. oryzae are shown. The sex locus is in grey. The tptA genes of Mcg and Mcc and the rnhA gene of Mcc (+) were not fully sequenced and are depicted in white with dotted outlines assuming conservation among the M. circinelloides subspecies. M.c. = Mucor circinelloides. (TIF) [file ppat.1002086.s006.tif]

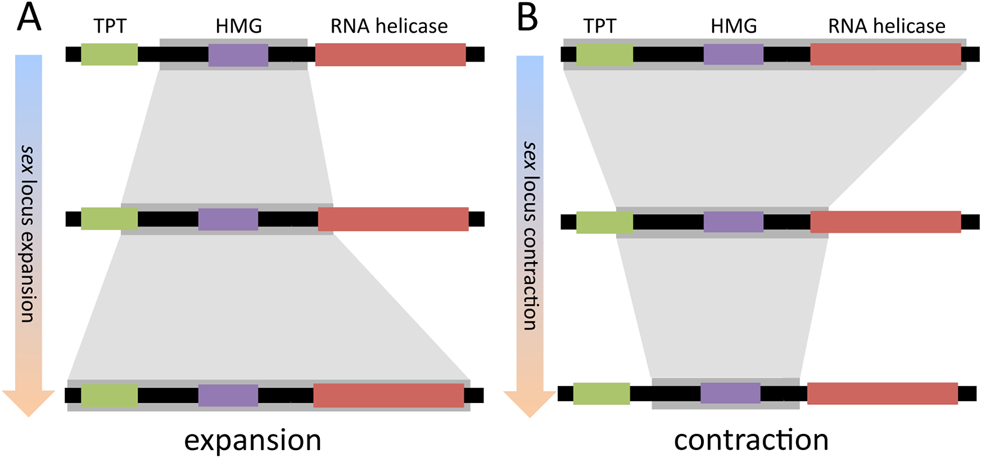

Supplement: Figure S7 — Hypothetical evolutionary trajectory of the sex locus. In the M. circinelloides complex, the sex locus could either expand (A) or contract (B) in size incorporating or evicting bordering genes or gene promoters as observed in other fungi (see text for details). Gene sizes are not to scale. (TIF) [file ppat.1002086.s007.tif]

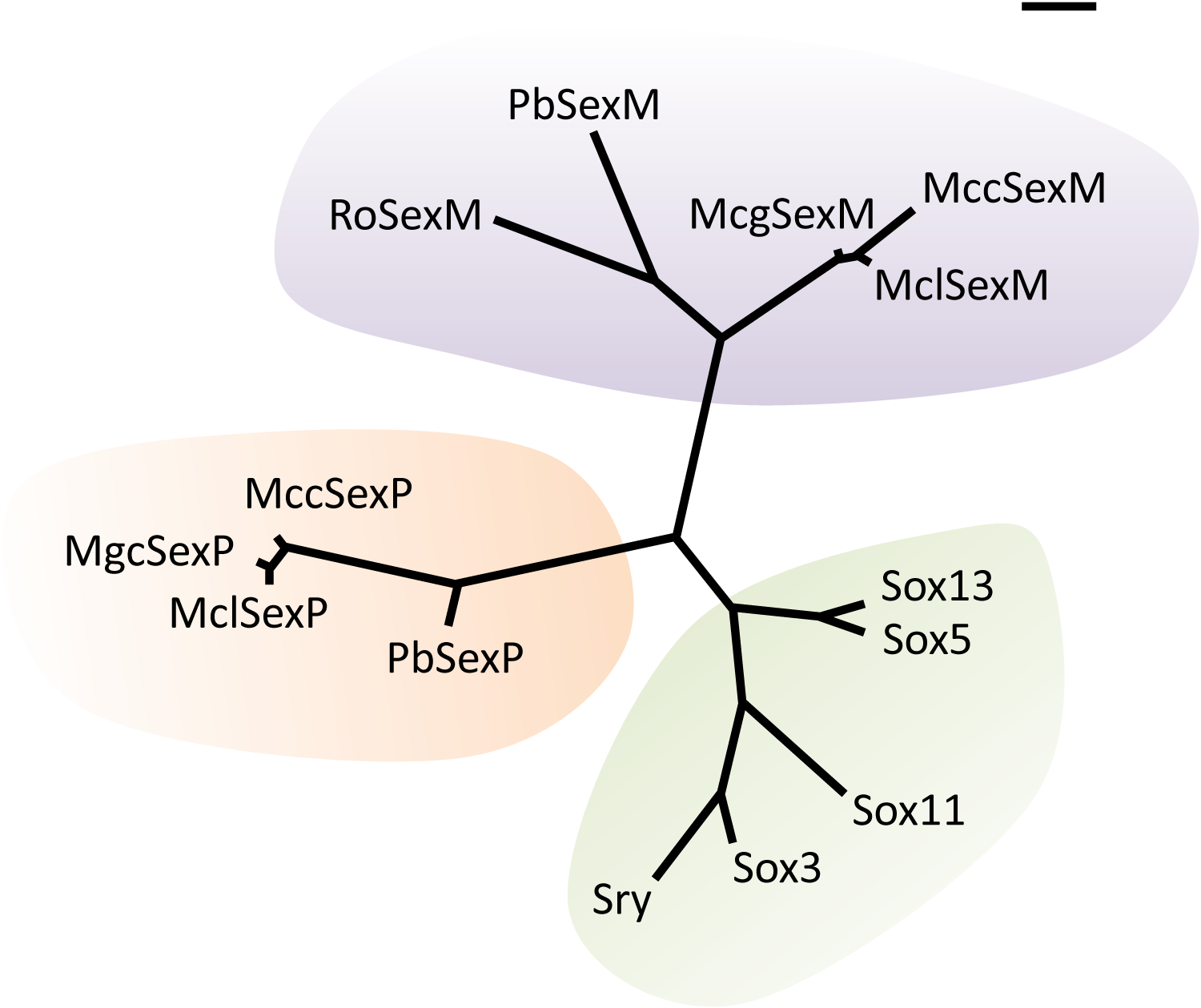

Supplement: Figure S8 — Unrooted phylogenetic tree for the SexP and SexM proteins. SexP proteins from different zygomycetes form a cluster, as do the SexM proteins, indicating that allelic sex determinant genes may have evolved before speciation within zygomycetes, especially in the Mucorales. Human HMG proteins (Sry, Sox3, Sox5, Sox11, and Sox13) were used as an outgroup. Pb: P. blakesleeanus, Ro: R. oryzae, Mcc: M. circinelloides f. circinelloides, Mcg: M. circinelloides f. griseocyanus, and Mcl: M. circinelloides f. lusitanicus. Scale = 0.5 (base pair substitution per base). (TIF) [file ppat.1002086.s008.tif]

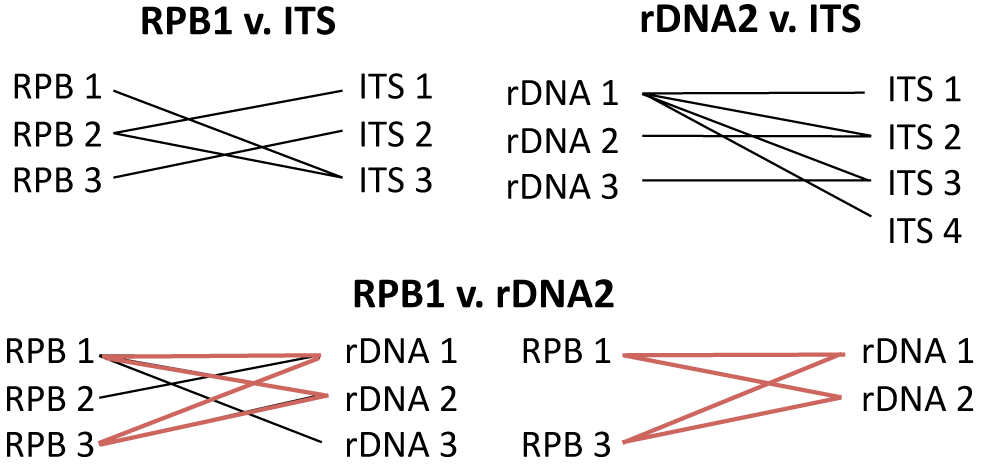

Supplement: Figure S9 — Possible sexual recombination within the clinical M. circinelloides isolates. Three loci, ITS, rDNA2, and RPB1, were examined to evaluate possible recombination within the Mcc species. Black lines indicate pairings of alleles within an isolate. Red lines highlight loci for which all four allele compatible combinations are observed (AB, ab, Ab, aB), providing evidence for recombination in the population. (TIF) [file ppat.1002086.s009.tif]
