# Supplementary material for: Sporangiospore Size Dimorphism Is Linked to Virulence of Mucor circinelloides
Source: PLoS Pathog. 2011 Jun 16;7(6):e1002086. doi: 10.1371/journal.ppat.1002086 (PMC3116813; doi:10.1371/journal.ppat.1002086)
Supplement: Table S1 — M. circinelloides f. lusitanicus strains used in this study. (DOC) [file ppat.1002086.s010.doc]

**Table S1**. *M. circinelloides* f. *lusitanicus* strains used in this study.

| Strains* | Mating type** | Sources | Lab Stock Number*** |
| --- | --- | --- | --- |
| CBS277.49 (CBS) | - | CBS | 9364 |
| CBS277.49 (AE) | - | Arturo Eslava's lab | 9363 |
| CBS277.49 (Murcia) | - | Santiago Torres-Martinez's lab | 9362/7412 |
| NRRL3631 (NRRL) | + | NRRL | 9366 |
| NRRL3631 (Murcia) | + | Santiago Torres-Martinez's lab | 9365/7413 |
| CBS852.71 | + | CBS | 9367 |
| CBS847.72 | - | CBS | 9368 |
| CBS108.17 | - | CBS | 9369 |
| CBS108.19 | - | CBS | 9370 |
| CBS242.33 | - | CBS | 9371 |
| CBS253.35 | - | CBS | 9372 |
| CBS633.65 | - | CBS | 9373 |
| CBS968.68 | + | CBS | 9374 |
| CBS969.68 | - | CBS | 9375 |
| ATCC1216a | - | ATCC | 9376/7415 |
| ATCC1216b | + | ATCC | 9377/7414 |

* Phylogenetic analysis with MLST confirmed that all strains listed are *M. circinelloides* f. *lusitanicus.*

** Mating type was confirmed by sexP/sexM specific PCR and mating assays.

*** The inventory number of the Heitman laboratory.
