# Supplementary material for: Sporangiospore Size Dimorphism Is Linked to Virulence of Mucor circinelloides
Source: PLoS Pathog. 2011 Jun 16;7(6):e1002086. doi: 10.1371/journal.ppat.1002086 (PMC3116813; doi:10.1371/journal.ppat.1002086)
Supplement: Table S2 — Primers used in this study. (DOC) [file ppat.1002086.s011.doc]

**Supplementary Table 2**. Primers used in this study.

| **Name** | **Sequence (5’🡪3’)** | **Remarks** |
| --- | --- | --- |
| JOHE19784 | TCCTCGACGCTTAAATTACCA | *sexM* disruption confirmation |
| JOHE19868 | ACCACATCACAATGGCAAAC | *sexM* disruption confirmation/*sex* locus sequencing |
| JOHE19869 | CTCGAGAAGCAGCGAGAAAT | *sex* locus PCR |
| JOHE19916 | ATTTCTCGCTGCTTCTCGAG | *sex* locus PCR |
| JOHE19917 | CGTTGAAGTTGGCAGTGATG | *sex* locus PCR |
| JOHE22785 | CTGGTGGTTCCAAGCTGTTT | *sex* locus PCR |
| JOHE22786 | TGCTTGCTCCATAGCTACACC | *sex* locus PCR |
| JOHE22787 | AGCGGTGAGAGCATACAAGG | *sex* locus PCR |
| JOHE22788 | GTAAGTATGCCCCGCGTTTA | *sex* locus PCR |
| JOHE22789 | CCTAAAGCGTGGTTCTCTGC | *sex* locus PCR |
| JOHE22790 | CAGTGCTTGTGAGCCTTCAA | *sex* locus PCR |
| JOHE20368 | CAATGCTGGAAAACACATGC | 5' end of *sexM* for disruption |
| JOHE20369 | CAAGTACCAATGCTGAGGCAATGCAGCTCAGGGGTTACAC | 5' end of *sexM* for disruption |
| JOHE20370 | GTGTAACCCCTGAGCTGCATTGCCTCAGCATTGGTACTTG | *pyrG* for *sexM* disruption cassette |
| JOHE20371 | GCAAATAACTCCTCGACGCTGTACACTGGCCATGCTATCG | *pyrG* for *sexM* disruption cassette |
| JOHE20372 | CGATAGCATGGCCAGTGTACAGCGTCGAGGAGTTATTTGC | 3' end of *sexM* for disruption |
| JOHE20373 | AGCTCGTTCGGCTGTACATT | 3' end of *sexM* for disruption |
| JOHE22452 | CCCTCAAAGACTTGG | RAPD primer |
| JOHE22453 | AATAGAACCCATCCC | RAPD primer |
| JOHE22460 | AACAACATCCATTATGC | RAPD primer |
| JOHE22461 | TGGCCTACCACGTTT | RAPD primer |
| P1 | GAGCACACGCATAGCATACG | *sexM* disruption confirmation |
| P2 | ATCTCAGCAGCATCGGATGG | *sexM* disruption confirmation |
