# Supplementary material for: Sporangiospore Size Dimorphism Is Linked to Virulence of Mucor circinelloides
Source: PLoS Pathog. 2011 Jun 16;7(6):e1002086. doi: 10.1371/journal.ppat.1002086 (PMC3116813; doi:10.1371/journal.ppat.1002086)
Supplement: Table S3 — GenBank accession numbers for ITS, rDNA2, RPB1, and sex locus. (DOC) [file ppat.1002086.s012.doc]

**Supplementary Table 3**. GenBank accession numbers for *ITS*, *rDNA2*, *RPB1*, and *sex* locus.

| **Species** | ***ITS*** | ***rDNA2*** | ***RPB1*** | ***sex* locus** |
| --- | --- | --- | --- | --- |
| *Mucor circinelloides* f. *circinelloides* (NRRL3614) (-) | HM754255 | HM754245 | HM754235 | HM754261 |
| *Mucor circinelloides* f. *circinelloides* (NRRL3615) (+) | HM754256 | HM754246 | HM754236 | HM754262 |
| *Mucor circinelloides* f. *circinelloides* (ATCC11010) (-) | HM754251 | HM754241 | HM754231 |  |
| *Mucor circinelloides* f. *circinelloides* (ATCC1209b) (-) | HM754254 | HM754244 | HM754234 |  |
| *Mucor circinelloides* f. *griseocyanus* (ATCC1207a) (+) | HM754252 | HM754242 | HM754232 | HM565940 |
| *Mucor circinelloides* f. *griseocyanus* (ATCC1207b) (-) | HM754253 | HM754243 | HM754233 | HM565941 |
| *Mucor circinelloides* f. *lusitanicus* (ATCC90680 or R7B) (-) | HM754260 | HM754250 | HM754240 | FJ009106 |
| *Mucor circinelloides* f. *lusitanicus* (NRRL3631) (+) | HM754259 | HM754249 | HM754239 | FJ009107 |
| *Mucor circinelloides* f. *lusitanicus* (ATCC1216b) (+) | HM754258 | HM754248 | HM754238 |  |
| *Mucor circinelloides* f. *lusitanicus* (ATCC1216a) (-) | HM754257 | HM754247 | HM754237 |  |
| *Mucor circinelloides* f. *lusitanicus* (CBS852.71) (+) | JF439683 | JF439674 | JF439665 |  |
| *Mucor circinelloides* f. *lusitanicus* (CBS847.72) (-) | JF439684 | JF439675 | JF439666 |  |
| *Mucor circinelloides* f. *lusitanicus* (CBS108.17) (-) | JF439685 | JF439676 | JF439667 |  |
| *Mucor circinelloides* f. *lusitanicus* (CBS108.19) (-) | JF439686 | JF439677 | JF439668 |  |
| *Mucor circinelloides* f. *lusitanicus* (CBS242.33) (-) | JF439687 | JF439678 | JF439669 |  |
| *Mucor circinelloides* f. *lusitanicus* (CBS253.36) (-) | JF439688 | JF439679 | JF439670 |  |
| *Mucor circinelloides* f. *lusitanicus* (CBS633.65) (-) | JF439689 | JF439680 | JF439671 |  |
| *Mucor circinelloides* f. *lusitanicus* (CBS968.68) (+) | JF439690 | JF439681 | JF439672 |  |
| *Mucor circinelloides* f. *lusitanicus* (CBS969.68) (-) | JF439691 | JF439682 | JF439673 |  |
| *Mucor circinelloides* f. *circinelloides* (Megoma) | HQ154612 | HQ179555 | HQ179564 |  |
| *Mucor circinelloides* f. *circinelloides* (CNRMA04.805) | HQ154608 | HQ179553 | HQ179560 |  |
| *Mucor circinelloides* f. *circinelloides* (CNRMA03.371) | HQ154607 | HQ179552 | HQ179559 |  |
| *Mucor circinelloides* f. *circinelloides* (CNRMA03.154) | HQ154606 | HQ179551 | HQ179558 |  |
| *Mucor circinelloides* f. *circinelloides* (IP1873.89) | HQ154605 | HQ179550 | HQ179565 |  |
| *Mucor circinelloides* f. *circinelloides* (CBS195.68) | HQ154604 | HQ179556 | HQ179557 |  |
| *Mucor circinelloides* (AS71) | HQ154610 | HQ179548 | HQ179562 |  |
| *Mucor circinelloides* (D-tate) | HQ154611 | HQ179549 | HQ179563 |  |
| *Mucor circinelloides* (UIC-1) | HQ154609 | HQ179554 | HQ179561 |  |
