# Supplementary material for: Sporangiospore Size Dimorphism Is Linked to Virulence of Mucor circinelloides
Source: PLoS Pathog. 2011 Jun 16;7(6):e1002086. doi: 10.1371/journal.ppat.1002086 (PMC3116813; doi:10.1371/journal.ppat.1002086)
Supplement: Text S1 — Strain typing with respect to CBS277.49, ATCC1216b, ATCC1216a, and NRRL3631. (DOC) [file ppat.1002086.s013.doc]

**Supplementary Text S1**

**Strain typing with respect to CBS277.49, ATCC1216b, ATCC1216a, and NRRL3631**

Based on the information available from the three different stock centers (ATCC, CBS, and NRRL), three of these isolates, CBS277.49 (the genome sequenced reference strain), ATCC1216b, and NRRL3631, were thought to be the same strain of (-) mating type that was originally from Dr. Blakeslee, but with different designations based on the repository source. While in a perfect world this should be the case, given the records available at the ATCC and CBS, in fact the three strains turn out to be three different isolates. This is based on both previous studies from other investigators, and our own analysis documenting differences in the mating type locus allele, mating type based on co-culture assays, and also MLST analysis. We expended considerable effort to resolve this issue definitively. Given that we received some of these strains directly from the culture collections, and others via well regarded collaborators in the field, we have contacted each of the stock centers (Teun Boekhout at CBS, enlisted now as an expert collaborator), ATCC, and NRRL (James Sweney) and received again each of these isolates directly from the stock centers for comparison with our lab stock isolates. In the MLST and *sex* locus sequence analysis and mating assays, two NRRL3631 isolates from Santiago Torres-Martinez’s lab and from the NRRL are indistinguishable from each other and three CBS277.49 isolates (Santiago Torres-Martinez’s lab, Arturo Eslava’s lab at the University of Salamanca, and CBS) are indistinguishable from each other. But NRRL3631 is clearly distinguishable from CBS277.49 as one is (+) mating type and the other (-) mating type.

Thus far, it is unequivocally clear that ATCC1216b, CBS277.49, and NRRL3631 are not all the same strain with different designations, but are in fact three different isolates of *Mucor circinelloides* f. *lusitanicus*. Two previous studies provided evidence that these strains may have different genetic backgrounds based on karyotype differences and mating type studies [1,2]. Our molecular analyses of the *sex* locus and mating assays document conclusively that these three isolates are not identical and while CBS277.49 is (-) mating type, ATCC1216b and NRRL3631 are both (+) mating type.

To further clarify these issues, we contacted experts in the field (Teun Boekhout, James Sweney, Santiago Torres-Martinez, June Kwon-Chung, and Arturo Eslava) to obtain information about the history and provenance of the strains, including information available from ATCC, NRRL and CBS, and found that ATCC1216b was deposited directly into ATCC by Dr. Albert F. Blakeslee himself, Carnegie Institute of Washington, Cold Spring Harbor, NY, on June 14, 1938. However, CBS277.49 was deposited into CBS by J. J. Harris in March 1949. Apparently there was more than a decade between the deposition of the two strains, and they were deposited by different individuals into two different stock centers, one in the US and the other in Europe. While they were at that time apparently thought to be the same isolate, that clearly isn’t the case, even though the species designation which is based on morphological characteristics, was correct, thankfully. It isn’t clear whether this mishandling occurred in the labs that prepared the isolates for deposition, or at the stock centers, and that may not be possible to attribute. In addition, when we originally purchased the ATCC1216b and ATCC1216a strains from the ATCC, we had found that ATCC1216a is (-) mating type and ATCC1216b is (+) mating type, which is reversed compared to their description in the ATCC and CBS databases. We have requested from the ATCC another independent culture of ATCC1216a and ATCC1216b, received these, and confirmed our original finding that ATCC1216a is (-) and ATCC1216b is (+), based on molecular and mating type analyses. Thus, the ATCC records are incorrect about the identity of these strains, and the strains were not inadvertently interchanged in our handling of them. In conclusion, the sequenced isolate CBS277.49 is (-) mating type and ATCC1216b and NRRL3631 are both (+) mating type, and thus they can’t all be the same isolate. We also found there are genomic sequence differences between the two (-) mating type strains, CBS277.49 and ATCC1216a, including SNP’s in the *RPB1* MLST locus and a different RAPD pattern, in addition to the karyotype difference identified by Nagy et al. (1994) and Diaz-Minguez et al. (1999) (Supplementary Text Figures 1 and 2 below). Thus, it doesn’t appear to be the case that the ATCC1216a and ATCC1216b isolates are simply interchanged in the ATCC stock collection, at least not based on their information that CBS277.49 and ATCC1216 were thought to be the same isolate (they are not since they differ in MLST gene sequences).

We codified the provenance of these strains in this study and have already been in touch with the three stock centers to assist them in clarifying their records. This is important for the field, as CBS277.49 is the isolate that was sequenced by the JGI (isolate initially obtained from CBS and genomic DNA purified and provided by our collaborator Santiago Torres-Martinez, who is the community coordinator for the *Mucor* genome project and a co-author and collaborator on this manuscript) and it will be important to ensure that anyone working in the field have the correct genome sequence isolate, as it is for those working on *Cryptococcus neoformans* to have bona fide H99 and those working on *Candida albicans* to have the authentic SC5314. It is a lesson for all of us that while the stock centers play an important role in storing isolates for >70 years, there is no substitute for very careful lab analysis and record keeping as fields and approaches advance.

We deposited all *Mcl* isolates included in this study in our lab strain collection and will make there available to anyone in the field upon request (Table 1S).

**References**

1. Nagy A, Vagvolgyi C, Balla E, Frenczy L (1994) Electrophoretic karyotype of *Mucor circinelloides*. Curr Genet 26: 45-48.

2. Diaz-Minguez JM, Lopez-Matas MA, Eslava AP (1999) Complementary mating types of *Mucor circinelloides* show electrophoretic karyotype heterogeneity. Curr Genet 36: 383-389.


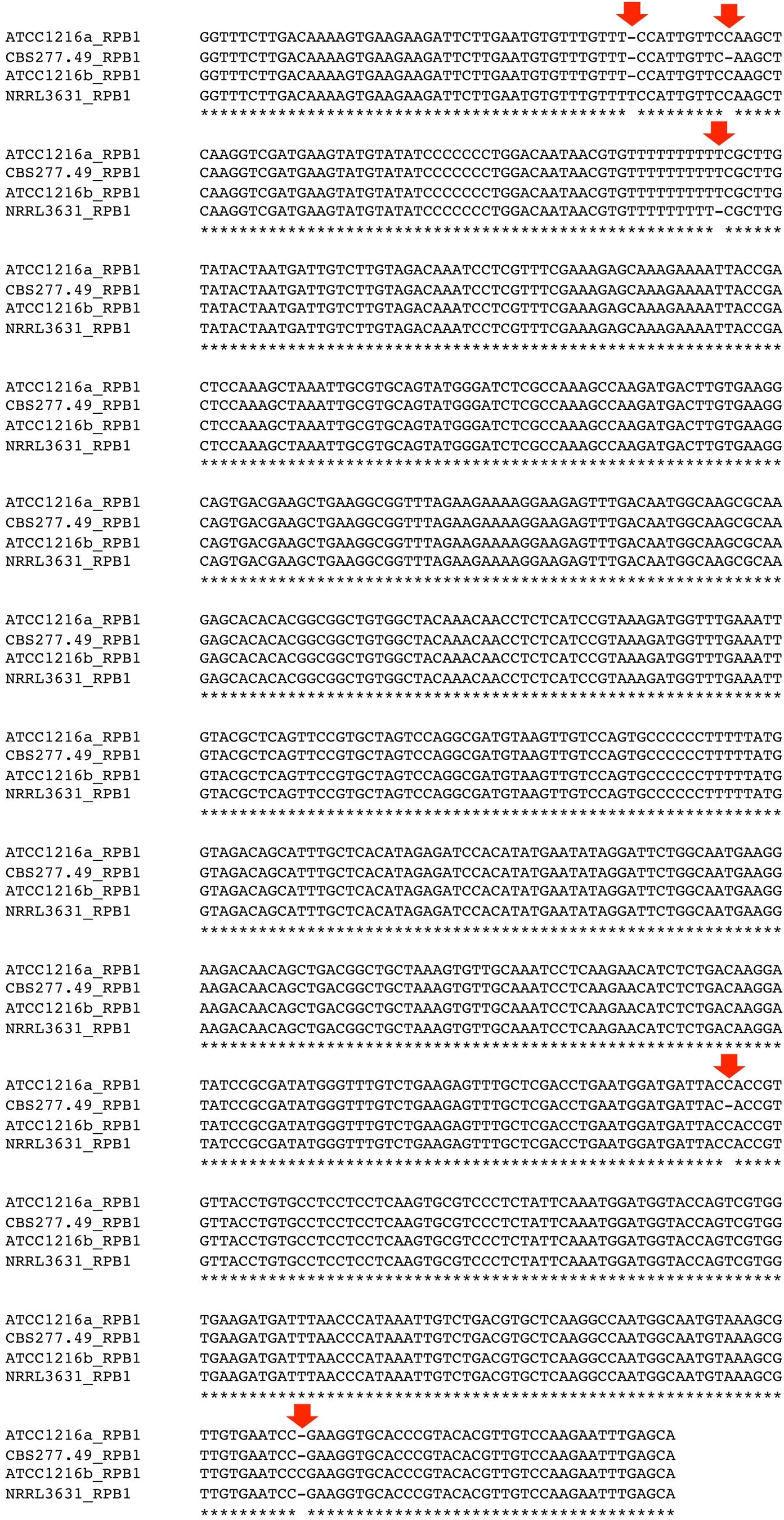


**Supplementary Text Figure 1**. Alignment of *RPB1* genes from CBS277.49, ATCC1216a, ATCC1216b, and NRRL3631. The *RPB1* gene was used in the phylogenetic analysis by MLST in this study. The DNA sequence alignment indicates each strain contains unique SNP’s suggesting they are all genetically different.


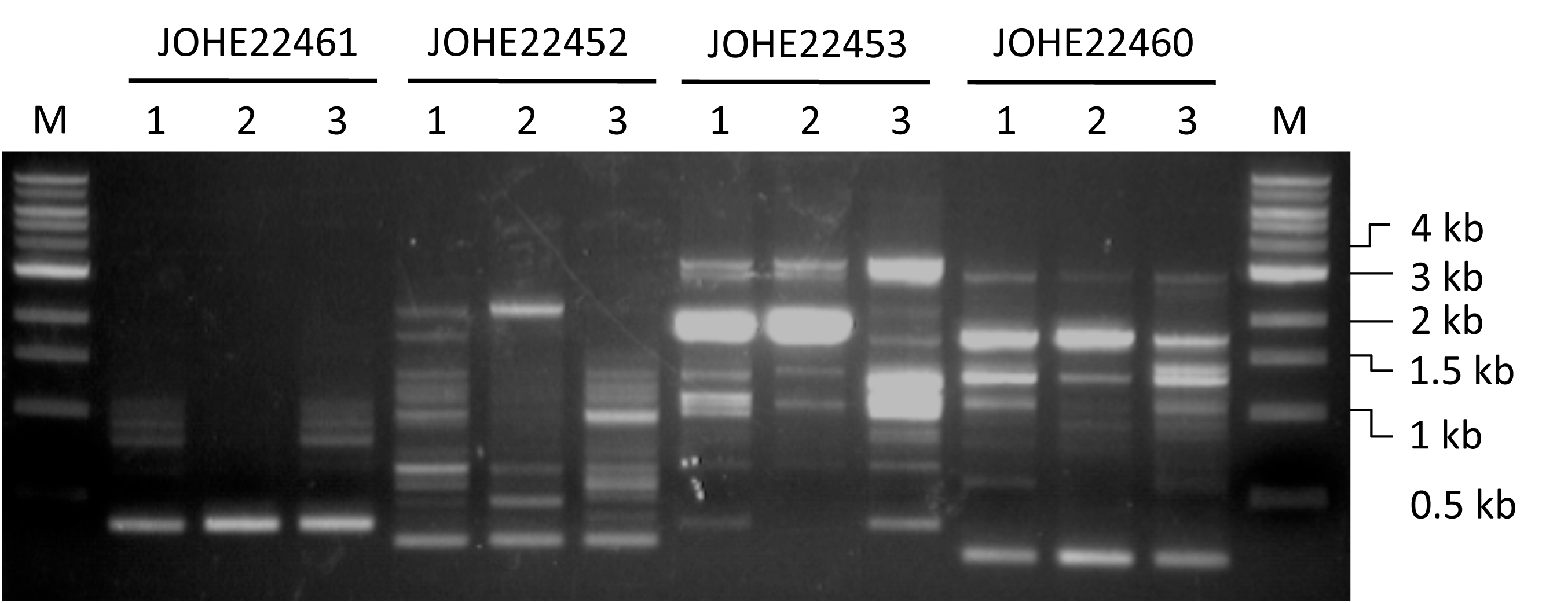


**Supplementary Text Figure 2**. RAPD patterns of CBS277.49, ATCC1216a, and NRRL3631. RAPD using four different primers (JOHE22461, JOHE22452, JOHE22453, and JOHE22460; see Supplementary Table 2) produces distinguishable banding patterns further suggesting those three strains are genetically distinct. 1: ATCC1216a, 2: CBS277.49, 3: NRRL3631. M: 1 kb DNA ladder marker (NEB). DNAs were separated on a 1% agarose gel.
